# Systematics and biology of Xylocopa subgenus Schonnherria (Hymenoptera, Apidae) in Argentina

**DOI:** 10.3897/zookeys.543.6300

**Published:** 2015-12-09

**Authors:** Mariano Lucia, Victor H. Gonzalez, Alberto H. Abrahamovich

**Affiliations:** 1 División Entomología, Museo de La Plata, Universidad Nacional de La Plata, Edificio Anexo Museo, Unidades de Investigación FCNyM, 122 y 60, 1900FWA, La Plata, Argentina. CONICET, Consejo Nacional de Investigaciones Científicas y Técnicas, Argentina; 2 Undergraduate Biology Program and Department of Ecology and Evolutionary Biology, Haworth Hall, 1200 Sunnyside Avenue, University of Kansas, Lawrence, Kansas, 66045, USA

**Keywords:** Anthophila, carpenter bees, host plants, nesting biology, pollinators, Xylocopini

## Abstract

Biological information on the species of the large carpenter bee Xylocopa subgenus Schonnherria occurring in Argentina is revised. Based on the appraisal of museum specimens, the study of type material, and field surveys conducted across 15 provinces between 2007 and 2011, the following seven species are recognized for the country: *Xylocopa bambusae* Schrottky, *Xylocopa chrysopoda* Schrottky, *Xylocopa macrops* Lepeletier de Saint Fargeau, *Xylocopa simillima* Smith *Xylocopa splendidula* Lepeletier de Saint Fargeau, *Xylocopa pulchra* Smith, and *Xylocopa viridis* Smith. Previous literature records of *Xylocopa dimidiata* Latreille, *Xylocopa subcyanea* Pérez, and *Xylocopa varians* Smith for the province of Misiones appear to have been misidentified specimens, although the presence of these species in Argentina cannot be entirely ruled out given the proximity of this province to Brazil and Paraguay where they occur; *Xylocopa boops* Maidl was described from a male specimen with unusually enlarged eyes and is newly synonymized under *Xylocopa macrops*. Males and females of all species are diagnosed, described, and figured, including details of the male genitalia. Taxonomic comments, data on the geographical distribution and nesting substrates, and identification keys to all Argentinean species of *Schonnherria* are provided. The nesting biologies of *Xylocopa splendidula* and *Xylocopa viridis* are documented.

## Introduction

Large carpenter bees of the genus *Xylocopa* Latreille (Apidae: Xylocopini) are conspicuous, commonly encountered, and fascinating elements of the melittofauna in many regions of the world. These usually robust, large, hairy bees nest in solid wood including structural timbers or in dead stalks of plants (hence their common name), althought some species nest in the ground. The more than 470 species are currently grouped in 31 subgenera, and approximately 100 species (in 12 subgenera) are recorded from the Neotropical region (Ospina 2000; Michener 2007; Moure 2007). The social behavior of *Xylocopa* is interesting and still poorly studied. Some species may live solitarily while others in semisocial or primitively eusocial nests, where the oldest female (mother or sister) feeds via trophallaxis both young females and males. Variation among polulations in these social behaviors may occur (Michener 1990). The diversity of mating behaviors is another interesting aspect of the biology of *Xylocopa*. For example, males in some species exhibit a lek-like mating behavior, defending areas that do not contain resources and at which females are attracted via pheromones. In other species, males patrol nesting or foraging sites and intercept females at flight (e.g., Gerling et al. 1989; Vinson and Frankie 1990; Prager and Richardson 2012). The male morphological modifications associated with such a diversity of mating behaviors are also outstanding. They range from the enlargement of the compound eyes, distinctly modified legs with a number of spines and protuberances of diverse shapes and sizes (Lucia et al. 2014), to the development of large pheromonal glands that change the configuration of the mesosoma (Minckley 1994).

Carpenter bees are also economically important. Some species are effective pollinators of diverse crops, including passion fruit, squash, tomato, Brazilian nut tree, and eggplant, and they are already being used for this purpose in different countries (e.g., Keasar 2010; Giannini et al. 2015). Additionally, some species such as *Xylocopa virginica* (Linnaeus) in North America or *Xylocopa frontalis* (Olivier) in South America may become an annoyance for people when nesting in houses or buildings. First, the large body size, the loud buzzing produced when flying or making their nests, and the approching behavior exhibited by males when patrolling a mating site, make these bees intimidating. Second, the various nest entrances and tunnels built inside the timber deface and may also weaken the nesting timber, thus causing structural damage and requiring control (Barrows 1980).

Despite the interesting biology, ecological importance and potential for development in crop pollination, most species of *Xylocopa* remain both biologically and taxonomically unknown. As for other taxa (Gonzalez et al. 2013), the diversity of *Xylocopa* is largely unexplored in many regions of the world and many species are difficult to nearly impossible to identify. Species from many areas are known only from the type specimen, a small number of specimes or from a single sex, the available descriptions do not allow for reliable identifications, and identification keys, when available, are poorly illustrated or not illustrated at all.

As part of an on-going effort to reduce such a taxonomic impediment and to increase our understanding of the biology of Neotropical carpenter bees, here we revise the species of Xylocopa subgenus Schonnherria Lepeletier de Saint Fargeau occurring in Argentina. This subgenus is the second most species-rich group of *Xylocopa* in the Western Hemisphere, containing about 30 species that range from southern United States to southern Argentina (Michener 2007). Here we recognized seven of the 11 species currently listed for the country (Hurd 1978); three of them appear to be records from misidentified specimens and one is placed in synonymy. We provide diagnoses, illustrations, descriptions, taxonomic comments, and information on the distribution to all species known to the country. This work is a continuation of a series of contributions dealing with the systematics and biology of carpenter bees in Argentina (Lucia and Abrahamovich 2010; Lucia et al. 2010; Ávalos-Hernández et al. 2011; Stuke et al. 2011; Lucia et al. 2012; Lucia and Gonzalez 2013; Lucia et al. 2014; Lucia et al. 2015).

## Material and methods

Morphological terminology generally follows that of Hurd and Moure (1963) and Michener (2007). The use of ventroapical plate of the male gonocoxite and lateral carina of the penis valve follows Michener (1975) and Minckley (1998); supraorbital line refers to the upper ocular tangent and is used here to indicate the position of the laterall ocelli in frontal view. External morphological features were studied using a Nikon SMZ 745T stereomicroscope. Photographs were taken with a Canon Power Shot® A520 digital camera attached to a steromicroscope and images were assembled using CombineZM open software (Hadley 2011). Species redescriptions emphasize structural characters, such as punctation, and are based on the examined specimens to include possible variations among characters. Ten specimens of each species were measured, except for *Xylocopa chrysopoda* Schrottky and *Xylocopa pulchra* Smith from which we were only able to measure two specimens of each. Measurements were taken with an ocular micrometer and were rounded to the nearest tenth of a millimeter; mean values and ranges are given in millimeters. Total body length was measured from the head to the apex of metasoma in lateral view; forewing length was measured at the anterior margin, from the apex of the costal esclerite to the wing apex; mesosoma width was measured between the outer borders of the tegulae; metasoma width was measured across the second tergum. The following abbreviations are used in the descriptions: T, S, F, and OD for metasomal terga and sterna, flagellar segments, and maximum diameter of the median ocellus, respectively. Male genitalia were mounted on metal studs and coated with gold-palladium for examination with a scanning microscope (SEM) Jeol-JSM-6360MV.

The primary types of all species treated herein were examined, as well as a total of 1702 specimens from all provinces of Argentina deposited in the following institutions and personal collections. The curators who kindly arranged the loans or allowed access to the collection in their care are indicated parentheses:

BMNH British Museum Natural History, London, UK (D. Notton)

CZ Private Collection of “Caire-Zelich”, Entre Ríos, Argentina (L. Caire-M. Zelich)

FAUBA Facultad de Agronomía Universidad de Buenos Aires, Buenos Aires, Argentina (J. P. Torretta)

IADIZA Instituto Argentino de Investigaciones de las Zonas Áridas, Mendoza, Argentina (G. Debandi)

IFML Fundación Miguel Lillo, San Miguel de Tucumán, Argentina (M.V. Colomo de Correa)

MACN Museo Argentino de Ciencias Naturales “Bernardino Rivadavia”, Buenos Aires, Argentina (A. Roig Alsina)

MLP Museo de La Plata, La Plata, Argentina (A. Lanteri)

MMP Museo Municipal de Ciencias Naturales “Lorenzo Scaglia”, Mar del Plata, Argentina (J. Farina)

MNCN Museo Nacional de Ciencias Naturales, Madrid, España (M. Paris)

MNHN Muséum National d’Histoire Naturelle, Paris, France (C. Villemant, A.Touret-Alby)

MZUSP Museu de Zoologia, Universidade de São Paulo, Brazil (R. Gonçalves)

NMW Naturhistorisches Museum Wein, Wein, Austria (D. Zimmermann-M. Vizek)

SEMC Snow Entomological Collection, Division of Entomology, University of Kansas Natural History Museum, Lawrence, Kansas, USA (M.S. Engel, J. Thomas, Z. Falin)

USNM National Museum of Natural History, Washington, DC, USA (B. Harris, S. Brady)

ZMB Museum für Naturkunde, Humbold-Universität zu Berlin, Berlin, Germany (F. Koch, V. Ritcher)

Information on the distribution was taken from literature and from specimen labels. The biogeographic provinces referred in the distribution account for each species followed those of Cabrera and Willink (1980). Complete label data for the examined material are available as supplemental material [online only]. Label data were transcribed literally, with bars “//” indicating information recorded on different labels.

Surveys for specimens as well as for nests of *Xylocopa* were conducted by M.L. between 2007 and 2011 across 15 provinces. The following provinces could not be sampled: Formosa, San Juan, La Pampa, Neuquén, Rio Negro, Santa Cruz, and Tierra del Fuego. When nests were found, the following variables were recorded: type of substrate and location (dead wood, structural timber, etc), plant species, and height above ground. Adults found inside nests were collected, killed, and deposited as vouchers in MLP. In the laboratory, the internal nest architecture was studied using three-dimensional molds made of liquid silicone rubber, as described in Lucia et al. (2014). Photographs of the nests were taken with a Panasonic® FZ18 digital camera.

Measurements of internal nest features were taken from the molds using a caliber. Mean values are provided with standard deviations. We used a Pearson correlation analysis to test for association between the following variables: tunnel length and tunnel width, total number of tunnels per nest and maximum diameter of the tree branch where the nest was found, total number of brood cells and total number of tunnels, and number of cells per number of tunnels and tunnel length.

## Results

### Systematics

#### Genus *Xylocopa* Latreille, 1802

##### Subgenus Schonnherria

Taxon classificationAnimaliaHymenopteraApidae

Lepeletier de Saint Fargeau, 1841

Xylocopa (Schonnherria) Lepeletier de Saint Fargeau, 1841: 207. Type species: *Xylocopa micans* Lepeletier de Saint Fargeau, 1841, by designation of Sandhouse, 1943: 598.
Xylocopa (*Schönherria*) Dalla Torre, 1896, 202, *lapsus calami pro Schonnherria* Lepeletier de Saint Fargeau, 1841 (not Burmeister, 1855: 417 [Coleoptera: Scarabaeoidea]).
Shornherria Ashmead, 1899: 71, error for *Schonnherria* Lepeletier de Saint Fargeau, 1841.
Xylocopa (Schoenherria) Hurd and Moure 1963: 118, *lapsus calami pro Schonnherria* Lepeletier de Saint Fargeau, 1841.
Xylocopa (Ioxylocopa) Hurd and Moure 1963: 116. Type species: *Xylocopa chrysopoda* Schrottky, 1902, by original designation; Minckley 1998: 36 [synonymy with *Schonnherria*].
Xylocopa (Xylocospila) Hurd and Moure 1963: 109. Type species: *Xylocopa bambusae* Schrottky, 1902, by original designation; Minckley 1998: 36 [synonymy with *Schonnherria*].

###### Diagnosis.

Species in the subgenus *Schonnherria* are small (15 mm) to moderately large bees (~24 mm), often with metallic highlights on all tagmata. *Schonnherria* can be distinguished from all other New World subgenera of *Xylocopa* by the following combination of characters: female mandible bidentate (except tridentate in *Xylocopa viridigastra* Lepeletier de Saint Fargeau, 1841), with apical tooth about as broad as or broader than basal tooth; T1 with complete gradulus on both sexes, remaining terga with gradulus absent (except in the male of *Xylocopa bambusae* with gradulus also on T2); male genitalia with a large spine of the ventral margin of the gonocoxite and the apex of the gonostylus bifid.

#### Species accounts

##### Xylocopa bambusae

Taxon classificationAnimaliaHymenopteraApidae

Schrottky, 1902

Figures 1 , 7 , 13 , 19 , 25 , 30 , 35 , 40 , 57

Xylocopa bambusae Schrottky, 1902: 475 (holotype: MZUSP; ♂, Rio Grande do Sul, Brazil) (examined)
Xylocopa eburnea Friese, 1903: 202 (lectotype: ZMB; ♂, Rio Grande do Sul, Brazil); Hurd and Moure 1961: 186 (lectotype designation and synonym with *bambusae*)
Xylocopa bellula Brèthes, 1916: 413 (holotype: MACN; ♀, Misiones, Argentina (examined); Hurd and Moure 1961: 187 (synonym with *bambusae* and unneccesary lectotype designation)
Xylocopa (Xylocospila) bambusae : Hurd and Moure 1963: 109.

###### Diagnosis.

**Figures 1–6.**
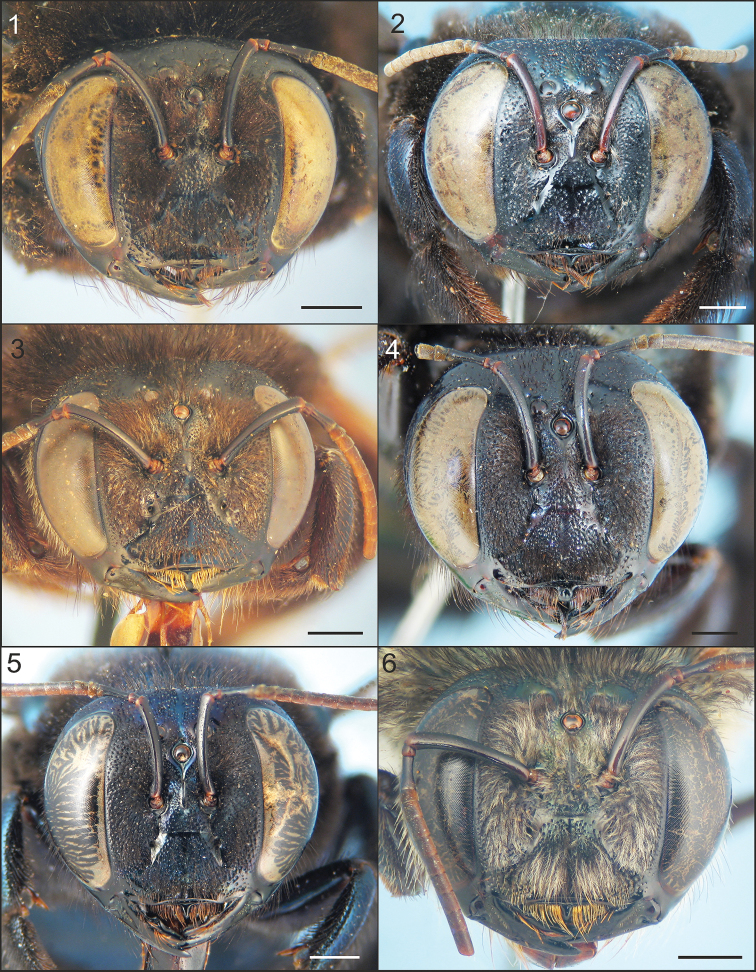
Faces of females of Xylocopa subgenus Schonnherria from Argentina. **1** *Xylocopa bambusae* **2** *Xylocopa macrops* **3** *Xylocopa pulchra* **4** *Xylocopa simillima* **5** *Xylocopa splendidula* **6** *Xylocopa viridis*. Scale bars: 1 mm.

**Figures 7–12.**
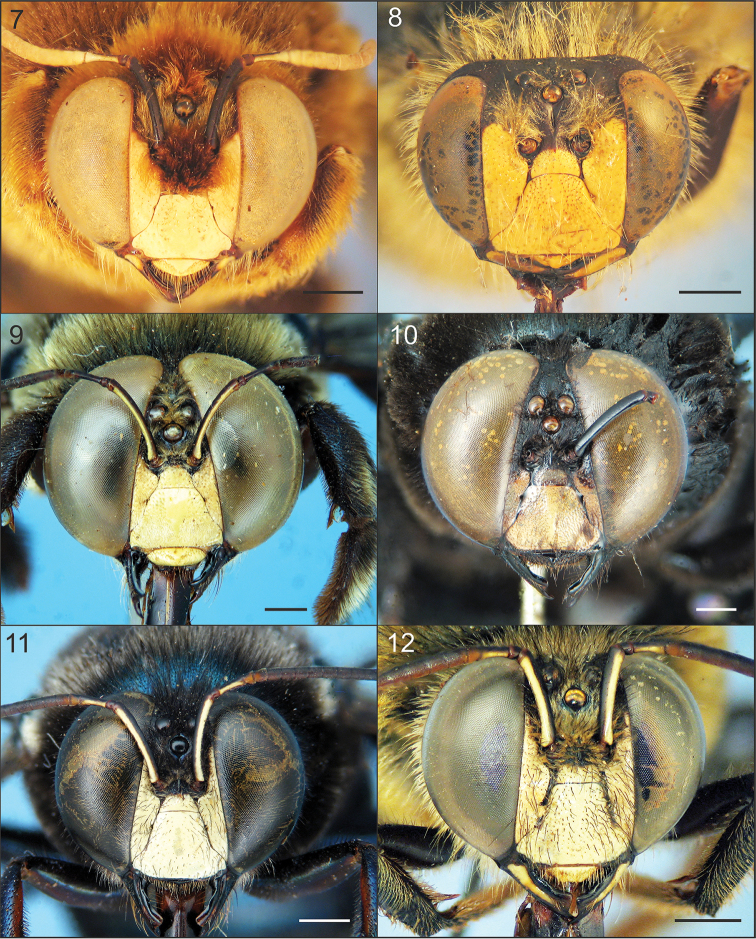
Faces of males of Xylocopa subgenus Schonnherria from Argentina. **7** *Xylocopa bambusae* **8** *Xylocopa chrysopoda* **9** *Xylocopa macrops* **10** *Xylocopa simillima* **11** *Xylocopa splendidula* **12** *Xylocopa viridis*. Scale bars: 1 mm.

**Figures 13–18.**
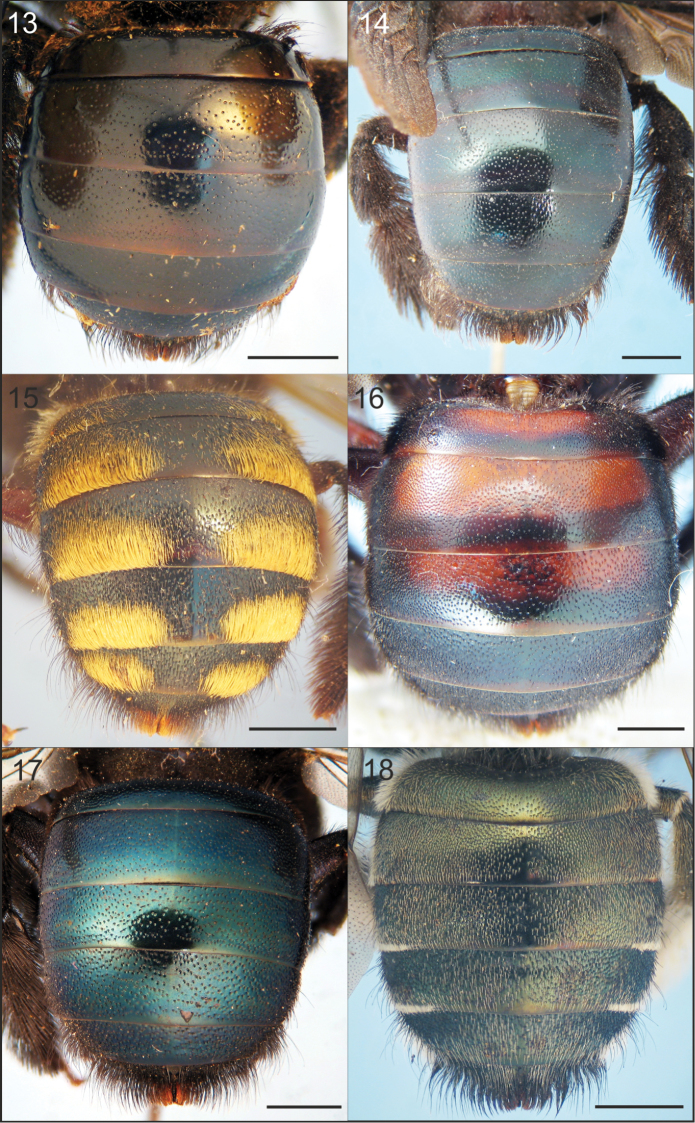
Dorsal view of metasoma of females of Xylocopa subgenus Schonnherria from Argentina. **13** *Xylocopa bambusae* **14** *Xylocopa macrops* **15** *Xylocopa pulchra* **16** *Xylocopa simillima* **17** *Xylocopa splendidula* **18** *Xylocopa viridis*. Scale bars: 2 mm.

**Figures 19–24.**
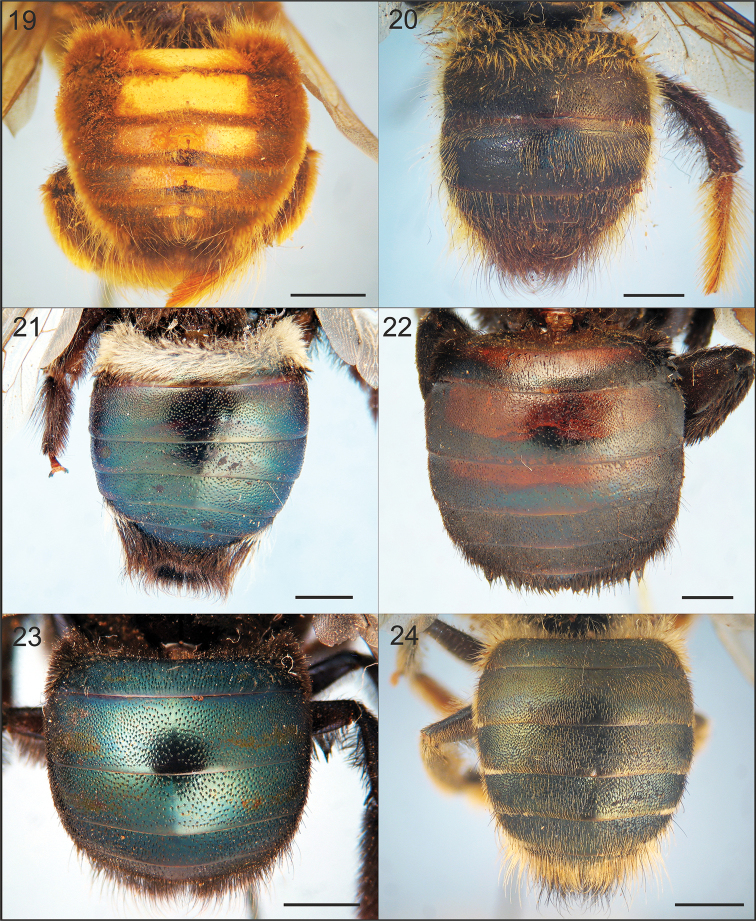
Dorsal view of metasoma of males of Xylocopa subgenus Schonnherria from Argentina. **19** *Xylocopa bambusae* **20** *Xylocopa chrysopoda* **21** *Xylocopa macrops* **22** *Xylocopa simillima* **23** *Xylocopa splendidula* **24** *Xylocopa viridis*. Scale bars: 2 mm.

The female of this species can be easily distinguished from other Argentinean species of *Schonnherria* by the following combination of characters: small body size (body length 14–17 mm); integument dark brown to black throughout, without metallic highlights; clypeus flat in profile, not elevated from adjacent paraocular area; clypeoalveollar distance long, about twice as long as longitudinal diameter of antennal socket; frontal carina projected into a distinct tubercle just above or at the upper tangent of anntenal sockets (Fig. 1); vertex and metasomal terga with large impunctate areas (Fig. 13); wings dark brown with weak coppery highlights basally, violet distally. The male can be easily recognized by the following combination of characters: small body size as in the female; body pubescence long, dense (obscuring integument in most areas), yellowish to reddish brown; supraclypeal area with a distinct tuft of long, erect, dense setae obscuring integument (Fig. 7); T1–T5 dark reddish brown, each with a distinct, broad, median yellow maculation on disc, sides and distal margins (inmaculate areas) densely covered by long, dense dark brown setae (Fig. 19). The female of this species superficially resembles *Xylocopa splendidula* and other small species of *Schonnherria* such as *Xylocopa lucida* Smith and *Xylocopa muscaria* (Fabricius) (not occurring in Argentina); however, those species have distinct metallic blue highlights on the body as well as different patterns in the punctation, pubescence, and wing coloration. The supraclypeal tuft of setae and pale maculations on the metasomal terga of the male of *Xylocopa bambusae* are so distinctive and unique among species of *Schonnherria* that these characters have been used to justify its recognition in a different subgenus.

###### Female.

Body length 15.9 (14.0–17.3); head length 4.2 (3.9–4.4); head width 4.9 (4.6–5.0); mesosoma width 5.7 (5.2–6.0); metasoma width 6.5 (5.8–6.8); forewing length 13.2 (12.3–13.7); forewing width 3.8 (3.6–4.0). *Coloration.* Integument dark reddish brown to black throughout, without metallic highlights, light brown on anterior face of F2–F9 and impunctate distal margins of metasomal terga and sterna. Wings dark brown with coppery highlights basally, golden iridescent. *Pubescence.* Dark brown to black. Head with scattered setae on vertex, gena, and clypeus distally (Fig. 1). Mesosoma with abundant, plumose setae except nearly asetose on discs of mesoscutum and mesoscutellum. T1 with scattered plumose setae on basal half, scattered, very short simple setae on distal half; T2–T4 medially with very short, simple setae, each seta barely exiting puncture; T3–T6 with long setae laterally; T5 with short (~1.0× OD), simple setae on disc; T6 with dense, long, both simple and plumose setae (Fig. 13); sterna with longer, denser, semierect setae than on terga. *Sculpturing.* Weakly imbricate to smooth and shiny. Vertex and upper gena largely impunctate, punctures small and scattered. Mesoscutum with scattered, coarse punctures on anterior margin and lateral to parapsidal lines, impunctate otherwise; mesoscutellum largely impunctate, with large, scattered punctures on disc. Metasomal terga with circular to ovoid punctures as large and coarse as those on mesoscutellum, most punctures separated by at least two times a puncture width, punctures denser laterally and on T5; sterna with elongate punctures on discs, punctures contiguous laterally, becoming sparser medially. *Structure.* Head broader than long (1.1–1.2:1); compound eyes with inner margins parallel; proportion of upper to lower interocular distance 1:1; middle interocular distance 2.9–3.0; vertex broad, distance between median ocellus and posterior margin of vertex 3.2–4.3 OD; orbitoccipital distance 0.75–0.85; lateral ocelli on supraorbital line; interocellar distance to ocelocular distance 1.0–1.3:1; interocellar to ocelloccipital 0.7–0.9:1; ocellocular to alveolocellar 0.6–0.7:1; orbitoccipital to ocellocular 1.1–1.3:1; alveolocular to interalveolar 0.9–1.1:1; clypeoalveolar distance long, about twice as long as longitudinal diameter of antennal socket; clypeocellar distance to distance between median ocellus and posterior margin of vertex 1.2–1.3:1; frontal carina projected into a distinct tubercle just above or at the upper tangent of anntenal sockets; clypeus broader than long, 2.0–2.4:1; proportion of length of scape, pedicel and F1–F4: 2.5–2.9:0.3–0.4:1:0.3–0.4:0.3–0.4:0.4, respectively; labrum broader than long, with three basal tubercles, median tubercle distinct, longitudinally enlongate, sublateral tubercles small, obscured by pubescence. Mesoscutellum gently convex; metanotum and propodeum obliquely inclined.

###### Male.

**Figures 25–30.**
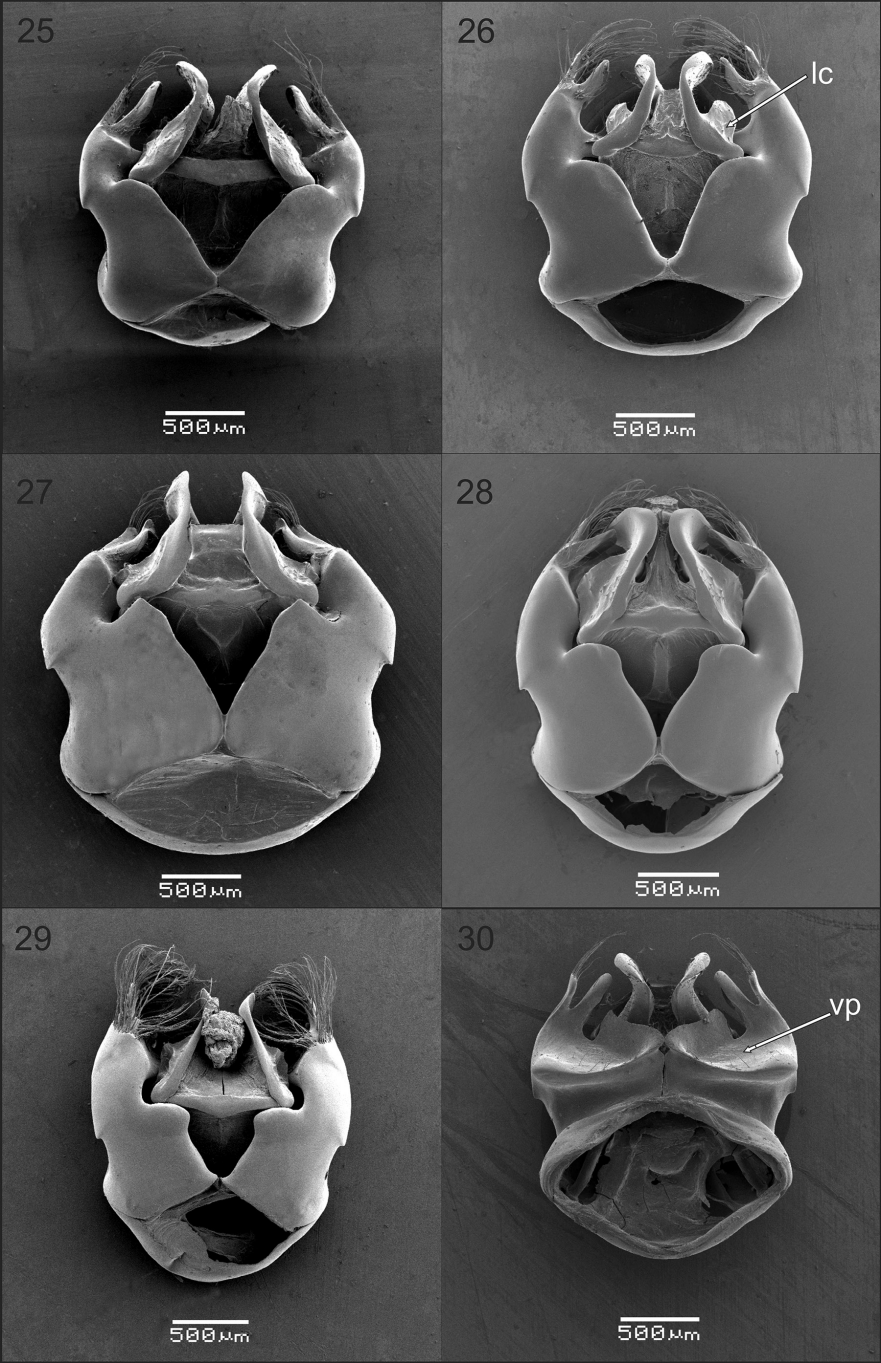
Genital capsule of males of Xylocopa subgenus Schonnherria from Argentina. All figures in dorsal view except Fig. **30** in ventral view. **25, 30** *Xylocopa bambusae* **26** *Xylocopa chrysopoda* **27** *Xylocopa macrops* **28** *Xylocopa splendidula* **29** *Xylocopa viridis*. Abbreviations: lc = lateral carina of penis valve; vp: ventroapical plate of gonocoxite.

As in the female, except for usual secondary sexual characters and as follows: body length 15.9 (15.0–16.7); head length 3.5 (3.5–3.6); head width 4.4 (4.4–4.6); mesosoma width 6.3 (6.0–6.6); metasoma width 6.6 (6.0–7.0); forewing length 11.6 (11.0–12.7); forewing width 3.6 (3.4–3.8). *Coloration.* Cream to yellowish maculations as follows: labrum, mandible basally, clypeus, supraclypeal area, paraocular area (except on upper one-third), anterior surface of flagellum (Fig. 7), outer surface of profemur basally, outer surfaces of protarsi, and discs of T1–T6 (maculations narrower on apical terga, sometimes absent on T4–T6) (Fig. 19). Wings subhyaline, yellowish with faint coppery highlights. *Pubescence.* Longer, denser than on female, yellowish to reddish brown, darker on face, vertex, sides of mesoscutum, mesoscutellum, metanotum, and inmaculate areas of terga (Fig. 19); supraclypeal area with a distinct tuft of long, erect, dense setae obscuring integument (Fig. 7); discs of mesoscutum and mesoscutellum asetose; maculated areas of terga with scattered erect setae. *Sculpturing.* Vertex and gena more densely punctate than in female, punctures separated by 1–2 times a puncture width; tegula impunctate, smooth and shiny on disc, otherwise dull, punctate (1–2 times a puncture width). Metasomal terga densely punctate (a puncture width or less) on inmaculated areas, including distal margins; punctures scattered on maculated areas. *Structure.* Middle interocular distance 2.0–2.1; distance between median ocellus and posterior margin of vertex 2.2–2.9 OD; orbitoccipital distance 0.4–0.6; interocellar to ocelocular distance 2.3–3:1; interocellar to ocelloccipital 1.2–1.4:1; ocellocular to alveolocellar 0.2–0.4:1; orbitoccipital to ocellocular 1.7–2.2:1; alveolocular to interalveolar 0.8–0.9:1; clypeoalveolar distance 1.5× longitudinal diameter of antennal socket; clypeocellar distance to distance between median ocellus and posterior margin of head 1.3–1.6:1; frontal carina strongly elevated, short 0.7–0.8, not tuberculiform as in female; clypeus broader than long, 1.5–1.6:1; proportion of length of scape, pedicel and F1–F4: 2.5–2.7:0.3–0.4:1:0.4:0.4:0.4. Mesoscutellum nearly flat, exposed, along same inclined plane with metanotum and base of propodeum; protrochanter with distinc spine; ventral surface of metatrochanter and metafemur basally glabrous, distinctly protuberant. Genitalia as in Figs 25, 30, 35, 40.

**Figures 31–34.**
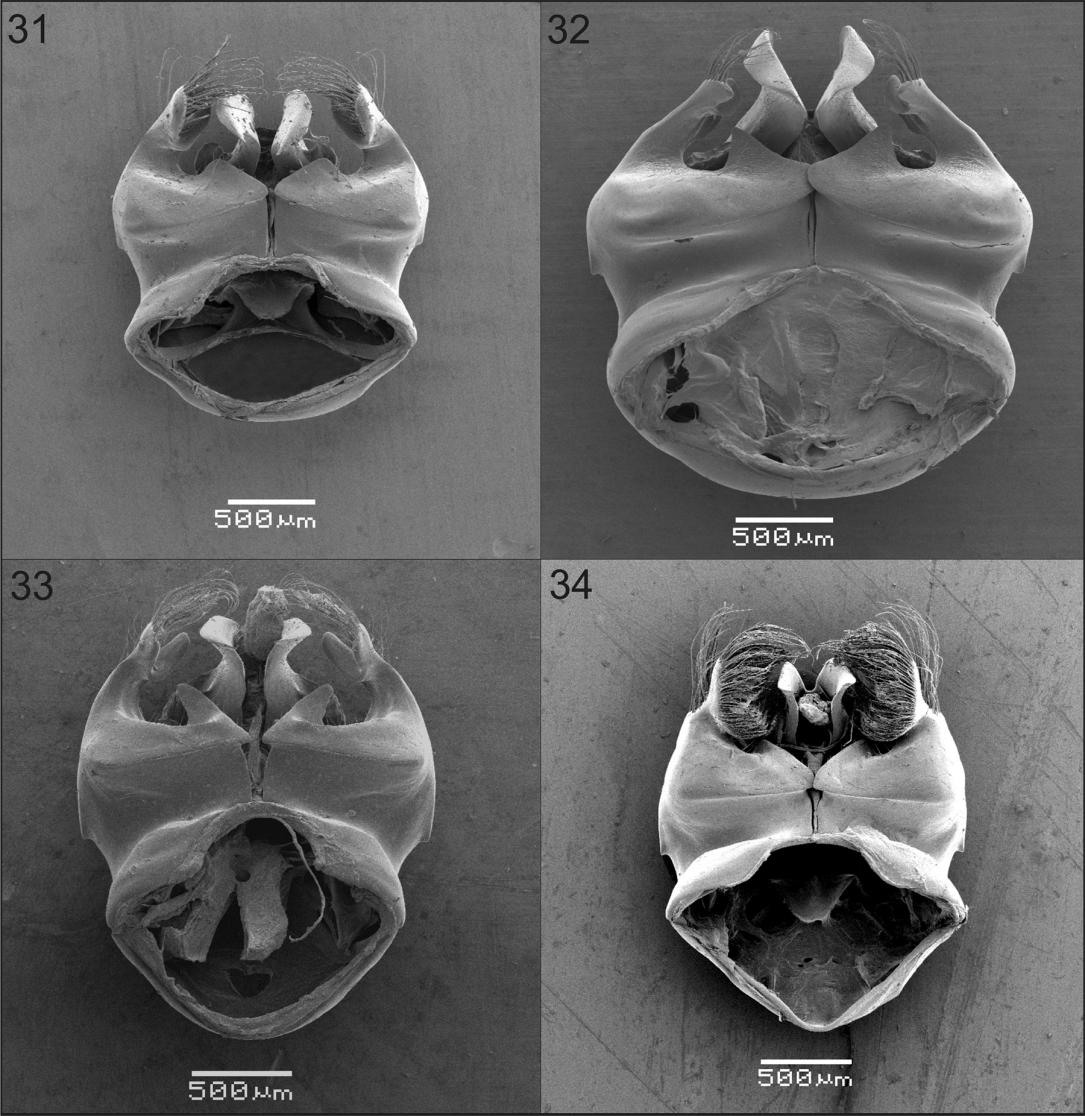
Ventral view of genital capsules of males of Xylocopa subgenus Schonnherria from Argentina. **31** *Xylocopa chrysopoda* **32** *Xylocopa macrops* **33** *Xylocopa splendidula* **34** *Xylocopa viridis*.

**Figures 35–44.**
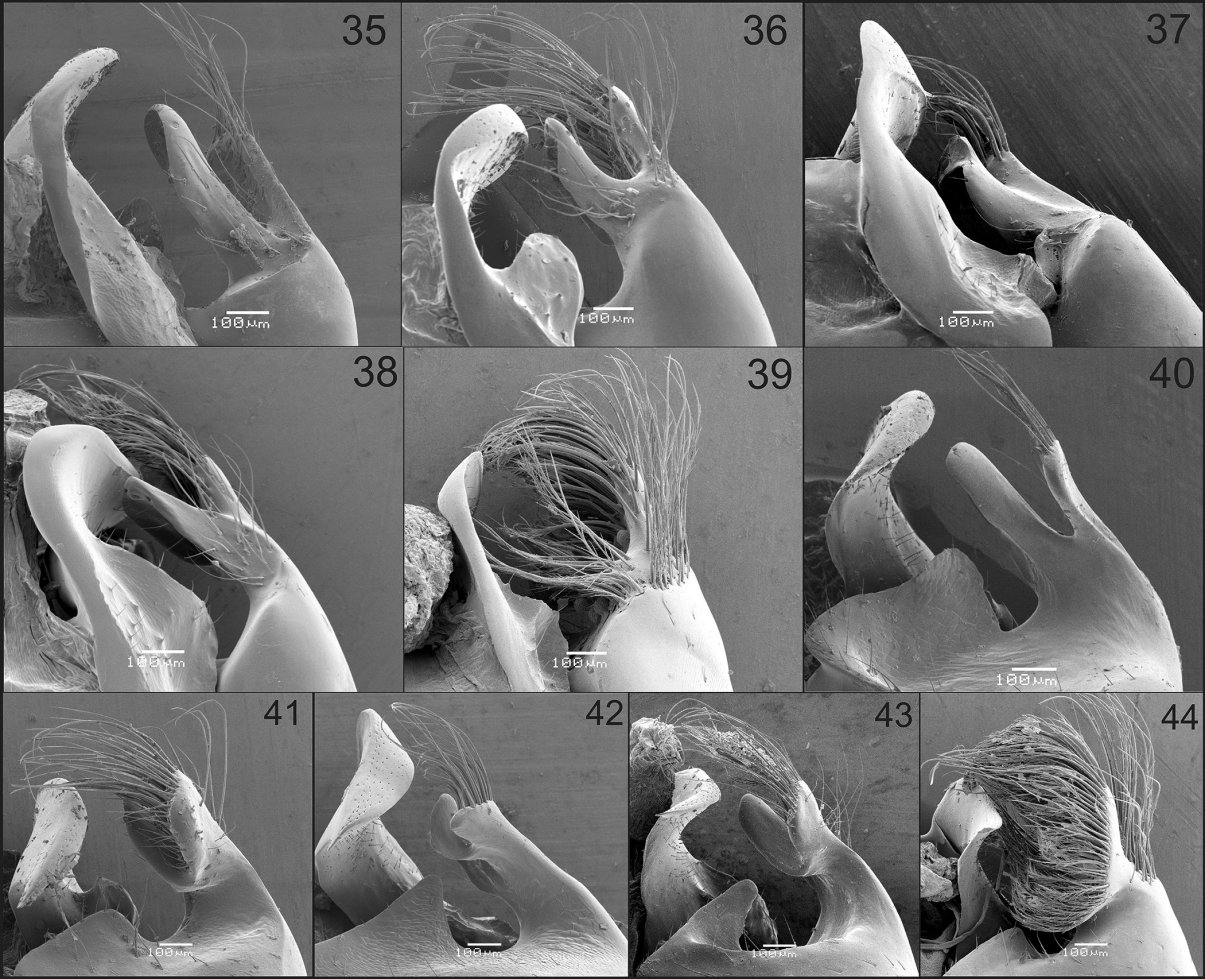
Dorsal (**35–39**) and ventral (**40–44**) views of apex of gonostylus and penis valve of males of Xylocopa subgenus Schonnherria from Argentina. **35, 40** *Xylocopa bambusae* **36, 41** *Xylocopa chrysopoda* **37, 42** *Xylocopa macrops* **38, 43** *Xylocopa splendidula* **39, 44** *Xylocopa viridis*.

###### Distribution.

This species is also known from Brazil and Paraguay (Table 1). In Argentina it has only been recorded from the province of Misiones, which is included in the Paranaense biogeographic province (Fig. 57).

**Table 1.** Summary of species of Xylocopa subgenus Schonnherria occuring in Argentina with information on their distribution. Province: BA = Buenos Aires; CA = Catamarca; CH = Chaco; CO = Córdoba; CT = Corrientes; ER = Entre Rios; FO = Formosa; JU = Juyuy; LP = La Pampa; LR = La Rioja; MZ = Mendoza; MI = Misiones; NQ = Neuquén; RN = Río Negro; SJ = San Juan; SL = San Luis; ST = Salta; SG = Santiago del Estero; SF = Santa Fe; TU = Tucumán. ? = province listed in the literature, but not confirmed in this study. General distributions were extracted from Hurd (1978), Moure (2007), and Montalva et al. (2008).

Species	General distribution	Distribution in Argentina	Altitude (m.a.s.l) in Argentina
*Xylocopa bambusae* Schrottky	Argentina, Brazil, Paraguay	MI	50–200
*Xylocopa chrysopoda* Schrottky	Argentina, Brazil	MI	170
*Xylocopa macrops* Lepeletier de Saint Fargeau	Argentina, Bolivia, Brazil, Colombia, Paraguay, Peru	BA, CH, CT, ER, FO, JU?, MI, SF, SG?, TU	0–1100
*Xylocopa pulchra* Smith	Argentina, Brazil, Colombia, Paraguay	MI, ST	170
*Xylocopa simillima* Smith	Argentina, Bolivia, Brazil, Paraguay, Peru	MI	170
*Xylocopa splendidula* Lepeletier de Saint Fargeau	Argentina, Brazil, Chile, Paraguay, Peru, Uruguay	BA, CA, CH, CH, CO, CT, ER, FO, JU, LP, LR, MZ, MI, NQ, RN, ST, SJ, SL, SF, SG, ST, TU	0–2700
*Xylocopa viridis* Smith	Argentina, Bolivia, Brazil, Colombia, Costa Rica, French Guiana, Guatemala, Guyana, Mexico, Panama, Paraguay, Peru	MI	170

###### Comments.

Hurd and Moure (1961: 186–187) designated a male and a female syntype as the lectotype of *Xylocopa eburnea* and *Xylocopa bellula*, respectively. In both cases Hurd and Moure (1961) unambiguously selected a specimen, provided information on the label data and repository collection, and clearly designated it as the sole name-bearing specimen, thus complying with the ICZN, Article 74. Such lectotypes designations appear to have been missed by Moure (2007: 673) who indicated syntypes for both species. The lectotype designation is valid for *Xylocopa eburnea* because Friese clearly mentions the existence of several males and females in the original description. However, the lectotype designation is unnecessary for *Xylocopa bellula* because Bréthes indicated a single female in the original description, which is currently housed at MACN. This specimen has a locality label that reads “Misiones, XII-1911, Abel Muniez”, a label in Bréthes’ handwriting and a holotype label [probably added recently]. Two other females, each bearing a locality label that reads “Misiones”, are also found with that specimen; one of them bears a lectotype label. Perhaps because of the additional two specimens, Moure and Hurd (1961) thought of them as syntypes and designated as lectotype the female collected on 1911. Curiously, the lectotype label is not found in that specimen but in one of the two additional females. To avoid further confusion, we added a red label with the following note to the type: ‘This is the true type as indicated in Bréthes’ (1913) original description. M. Lucia & V.H. Gonzalez 2015’.

Despite actively searching for nests as well as specimens of this species in insect collections and in the field, the only specimens available to us were those collected by Peter Jörgensen at different times of the year in Misiones during the first decade of 1900 (see supplemental material). The species has not been collected ever since. The nesting biology of this *Xylocopa bambusae* is unknown, although it presumably nests in bamboo stalks (Schrottky 1902).

##### Xylocopa chrysopoda

Taxon classificationAnimaliaHymenopteraApidae

Schrottky, 1902

Figures 8 , 20 , 26 , 31 , 36 , 41 , 58

Xylocopa chrysopoda Schrottky, 1901: 214 (*nomen nudum*)
Xylocopa chrysopoda Schrottky, 1902: 475 (holotype: MZUSP; ♂, Jundiaí, São Paulo, Brazil) (examined)
Xylocopa (Ioxylocopa) chrysopoda : Hurd and Moure 1963: 116.

###### Diagnosis.

This species is known only from the male. It can be distinguished from other Argentinean species of *Schonnherria* by the following combination of characters: small body size (body length 17.0–17.7); integument dark brown to black throughout, at most with faint metallic highliths on metasomal terga; compound eyes not enlarged, parallel or nearly so (Fig. 8); metasomal terga uniformly punctate, punctures separated by 1–2 times a puncture width, with distinctly long (≥ 2.0 OD) setae on discs (Fig. 20).

###### Male.

Body length 17.3 (17.0–17.7); head length 3.5 (3.5–3.6); head width 4.4; mesosoma width 6.0; metasoma width 6.7 (6.6–6.8); forewing length 14; forewing width 3.5 (3.4–3.6). *Coloration.* Integument dark brown to black except tarsi light reddish brown and cream to yellow maculations as follows: outer surface of mandible (except margins), clypeus, supraclypeal area basally, and paraocular area (except on upper one-third) (Fig. 8). Wings sybhyaline, yellowish, with weak coppery highlights. *Pubescence.* Long, dense, predominantly yellowish, setae dark brown to black on vertex, metafemur basally, dorsal surface of metatibia, and discs of T2–T7 (variably mixed with yellowish setae) (Fig. 20). *Sculpturing.* Integument largely imbricate among punctures, nearly smooth and shiny on outer surface of mandible, labrum, maculate areas of face, and discs of mesoscutum and mesocutellum. Upper paraocular area, frons, and interocellar area with coarse, contiguous punctures; vertex and gena with punctures sparser than on upper paraocular area. Discs of mesoscutum and mesoscutellum largely impunctate, otherwise punctures shallower than and about as dense as those on vertex. Discs of metasomal terga uniformly punctate, setiferous punctures circular to ovoid, separated by 1–2 times a puncture width, distal margins impunctate (Fig. 20); sterna more densely punctate than on terga. *Structure.* Head broader than long (1.2–1.3:1); compound eyes not enlarged, parallel or nearly so (Fig. 8); proportion of upper to lower interocular distance 0.9–1:1; upper interocular distance 6.3–6.9× OD; middle interocular distance 2.60; distance between median ocellus and posterior margin of vertex 2.6–2.7 OD; orbitoccipital distance 0.60; lateral ocelli on supraorbital line; interocellar distance to ocelocular distance 1.2–1.3:1; interocellar to ocelloccipital 0.5–1.1:1; ocellocular to alveolocellar 0.5–0.6:1; orbitoccipital to ocellocular 1.2–1.5:1; alveolocular to interalveolar 1.1–1.2:1; clypeoalveolar distance 1.5 times longitudinal diameter of antennal socket; clypeocellar distance to distance between median ocellus and posterior margin of head 1.4–1.5:1; frontal carina elevated, short 0.8–0.9; clypeus broader than long, 0.6:1; proportion of length of scape, pedicel and F1–F4: 2.3:0.4:1:0.4:0.5:0.6, respectively. Mesoscutellum nearly flat, exposed, along same inclined plane with metanotum and base of propodeum; metatibia apically swollen on inner surface. Genitalia as in Figs 26, 31, 36, 41.

###### Female.

Unknown.

###### Distribution.

This species is known only from Brazil and the province of Misiones in Argentina, the latter area included in the Paranaense biogeographic province (Fig. 58).

###### Comments.

Schrottky (1912) suggested that *Xylocopa chrysopoda* may be the male of *Xylocopa pulchra*, a species currently known from the female sex. As for *Xylocopa bambusae*, these two species are rarely collected and are currently known from a limited number of specimens. We did not collect nor examine specimens of *Xylocopa chrysopoda* from Argentina captured in the last 100 years.

##### Xylocopa macrops

Taxon classificationAnimaliaHymenopteraApidae

Lepeletier de Saint Fargeau, 1841

Figures 2 , 9 , 14 , 21 , 27 , 32 , 37 , 42 , 55

Xylocopa macrops Lepeletier de Saint Fargeau, 1841: 209 (holotype: ♂, Brazil, whereabouts unknown)
Xylocopa crotalariae Schrottky, 1901: 212, 214 (*nomen nudum*)
Xylocopa crotalariae Schrottky, 1902: 472 (holotype: MZUSP; ♀, Jundiaí, São Paulo, Brazil) (examined); Hurd and Moure 1963: 302 (synonym with *macrops*)
Xylocopa boops Maidl, 1912: 325 (holotype: NMW; ♂, Brazil) (examined, **syn. n.**)
Xylocopa (Schonnherria) macrops : Hurd and Moure 1963: 302

###### Diagnosis.

The female of this species can be distinguished from other Argentinean species of *Schonnherria* by the following combination of characters: medium-sized bees (18–22 mm); body pubescence black; integument black throughout with very weak metallic blue, often most evident on mesoscutum, tegula, outer surfaces of pro- and mesotibiae, and metasomal terga; clypeus depressed basally on disc, dorsolaterally elevated from adjacent paraocular area (Fig. 2); vertex, behind lateral ocelli, with coarse punctures separated by 1–2 times a puncture width; metasomal terga sparsely punctate, punctures coarse and separated by at least two times a puncture width (Fig. 14); wings black with metallic green and violet highlights. The male can be easily recognized by the following combination of characters: compound eyes enlarged, strongly convergent dorsally (Fig. 9); meso- and metasoma with distinct metallic blue highlights; metasoma sparsely punctate with very short setae, each seta barely exiting puncture; discs of S2–S6 each with sublateral yellow maculation. The female of this species superficially resembles that of *Xylocopa simillima* and *Xylocopa dimidiata* Latreille (not occurring in Argentina, see below) in the body size and black color of the integument, pubescence and wings. However, it can be separated from both species primarily by the midbasal tubercle of the labrum and the shape of clypeus. In those species the labrum has a single, large, capitate tubercle and the disc of the clypeus is flat. In *Xylocopa macrops* the labrum has three tubercles, a longitudinally elongated median tubercle and two small sublateral tubercles, and the clypeus is basally depressed, dorsolaterally elevated from adjacent paraocupar area. The male can be confused with that of *Xylocopa splendidula* by the compound eyes strongly convergent dorsally and the blue metallic highlights of the metasoma. However, in *Xylocopa macrops* the metafemur is distinctly modified, with the ventral margin strongly protuberant, the metabasitarsus is robust and shorter than the metatibia. In *Xylocopa splendidula* both the metafemur and metabasitarsus are unmodified, and the latter is longer than the metatibia; also, yellow maculations are absent from the labrum and discs of the sterna (present in *Xylocopa macrops*).

###### Female.

Body length 19.6 (18.3–21.7); head length 5.1 (4.8–5.4); head width 6.1 (5.8–6.4); mesosoma width 6.6 (6.2–7.0); metasoma width 7.6 (7.2–8.0); forewing length 15.9 (15.0–17.3); forewing width 4.8 (4.4–5.4). *Coloration.* Integument dark reddish brown to black with weak metallic blue-green highlights, particularly on mesoscutum and metasomal terga. Wing dark brown to black with metallic violet-green highlights. *Pubescence.* Predominantly black, except by pale setae intermixed with black setae on face and sides of T4–T6 and S4–S6 (sometimes absent). Head with abundant setae, scattered on gena. Mesosoma with abundant, plumose setae, nearly asetose on discs of mesoscutum and mesoscutellum. T1 with plumose setae on basal half, with very short simple setae on distal half, each seta barely exiting puncture; T2–T5 with short setae as on distal half of T1, setae longer and denser on sides of T5 and T6 (Fig. 14); sterna with longer setae than on terga, setae progressively becoming denser and longer towards apical segments. *Sculpturing.* Weakly imbricate to smooth and shiny. Clypeus with dorsolateral elevated area impunctate; vertex sparsely punctate, punctures denser behind lateral ocellus (1–2 times a puncture width), becoming sparser and shallower on upper gena. Mesoscutum with coarse, sparse punctures as on vertex along anterior margin and lateral to pasapsidal lines, impunctate otherwise; mesoscutellum largely impunctate on anterior half. Metasomal terga with circular to ovoid punctures smaller than those on posterior half of mesoscutellum, punctures separated by 2–3 times a puncture width, denser and elongated on T5 (Fig. 14). *Structure.* Head broader than long (1.1–1.3:1); compound eyes with inner margins parallel; proportion of upper to lower interocular distance 0.8–0.9:1; middle interocular distance 3.7–4.0; vertex broad, distance between median ocellus and posterior margin of vertex 3.7–4.1 times OD; orbitoccipital distance 0.7–0.9; lateral ocelli below supraorbital line; interocellar distance to ocelocular distance 0.8–1.0:1; interocellar to ocelloccipital 0.5–0.6:1; ocellocular to alveolocellar 0.6–0.8:1; orbitoccipital to ocellocular 0.8–1:1; alveolocular to interalveolar 1.0–1.2:1; clypeoalveolar distance short, about as long as longitudinal diameter of antennal socket; clypeocellar distance to distance between median ocellus and posterior margin of vertex 0.8–1:1; frontal carina elevated and forming a low tubercle near lower tangent of antennal sockets, continuing dorsally into a low carina (Fig. 2); clypeus broader than long, 2.1–2.3:1, depressed basally on disc, dorsolaterally elevated from adjacent paraocular area; supraclypeal area depressed on disc, laterally elevated from adjacent paraocular area; proportion of length of scape, pedicel and F1–F4: 2.9–3.5:0.3–0.4:1:0.3–0.4: 0.4–0.5:0.4–0.5, respectively; labrum broader than long, with three basal tubercles, median tubercle distinct, longitudinally elongate, sublateral tubercles small, obscured by pubescence. Mesoscutellum gently convex; metanotum and propodeum vertical.

###### Male.

As in the female, except for usual secondary sexual characters and as follows: body length 21.6 (20.7–22.7); head length 5.1 (4.9–5.4); head width 6.2 (6.0–6.4); mesosoma width 7.3 (7.0–7.6); metasoma width 7.7 (7.2–8.0); forewing length 15.5 (14.3–16.0); forewing width 4.1 (4.0–4.4). *Coloration.* Integument with distinct metallic blue-green highlights. Cream to yellow maculations as follows: labrum, mandible basally (sometimes absent), clypeus, supraclypeal area, inferior paraocular area, anterior surface of scape, and discs of S2–S6 each with sublateral, triangular maculation (sometimes absent on S2, S5 and S6). Wings hyaline to subhyaline with weak golden highlights. *Pubescense.* Longer and denser than on female, predominantly whitish, except dark brown to black on: interocelar area, vertex, pronotal lobe, metepisternum, propodeum, procoxa, inner surfaces of profemur and protarsi, remaining legs excluding posterior margin of metatibia, sides of T1, T5, T6, sides of sterna, and discs of S4–S6. White pubescence on T1 dense, nearly obscuring integument. *Sculpturing.* Coarser and denser than in female, particularly on sides of mesoscutum and mesoscutellum, and terga (Fig. 21). *Structure.* Compound eyes strongly converging dorsally, proportion of upper to lower interocular distance 0.1–0.2:1; upper interocular distance 0.5–0.8 times OD; middle interocular distance 2.0–2.2; distance between median ocellus and posterior margin of vertex 3.6–4.2 OD; orbitoccipital distance absent due to enlargement of eyes; lateral ocelli well below supraorbital line; interocellar distance to ocelocular distance 4.0–9.0:1; interocellar to ocelloccipital 0.2–0.3:1; ocellocular to alveolocellar 0.1:1; orbitoccipital to ocellocular reduced, alveolocular to interalveolar 0.4–0.6:1; clypeocellar distance to distance between median ocellus and posterior margin of vertex 0.5–0.6:1; clypeus broader than long, 1.5–1.7:1; proportion of length of scape, pedicel and F1–F4: 3.0–3.2:0.3–0.4:1:0.2–0.3:0.3–0.4:0.4–0.5, respectively. Legs robust; mesobasitarsus shorter than mesotibia; metacoxa and metatrochanter toothed; metafemur protuberant ventrally; metatibia with distinct cavity distally on inner surface bordered by two spinous projections, inner projection triangular, short, outer projection broader than and about as long as tibial spur; metabasitarsus robus, shorter than metatibia. Genitalia as in Figs 27, 32, 37, 42.

###### Distribution.

This species appears to be widely distributed in South America, occuring from Colombia to Brazil (Table 1). We examined or collected specimens from the following nine provinces in Argentina (Fig. 55): Buenos Aires, Chaco, Corrientes, Entre Ríos, Formosa, Misiones, Salta, Santa Fe, and Tucumán. Hurd (1978) also recorded this species from Jujuy and Santiago del Estero. It occurs in the following biogeographic provinces: Chaqueña, Pampeana, Paranaense, and Yungas.

###### Comments.

Maidl (1912) described *Xylocopa boops* based on a single male from an unspecified location in Brazil. The species was later listed by Hurd (1978) from Tafi, Province of Tucuman, Argentina. We examined the type specimen currently deposited at NMW (Figs 49–52) as well as the male specimen from Argentina deposited at USNM. Both specimens closely agree with *Xylocopa macrops* in all morphologically external characters, including the genitalia, except by the size of their compound eyes. In these specimens the eyes are extremely large and convergent above so that their upper margins are nearly in contact dorsally (Figs 49, 50). Such upper interorbital distance is about 0.23 times OD in the type of *Xylocopa boops* whereas it ranges from 0.54–0.72 times OD in specimens of *Xylocopa macrops* (*n* = 10). The shapes of the gonocoxite, apex of gonostylus, and lateral carina of the penis valve, which have proven to be reliable in species recognition in *Schonnherria* (Figs 25–44), are identical between *Xylocopa boops* and *Xylocopa macrops*. Thus, it seems to us that *Xylocopa boops* was described from an individual of *Xylocopa macrops* with unusually large eyes and we have decided not to recognize this species. Here it is synonymized under *Xylocopa macrops*.

Hurd (1978: 25) listed *Xylocopa subcyanea* Pérez from Misiones and Torreta et al. (2010) from Paraná, province of Entre Rios. We were not able to capture or find any specimen of this species from Argentina in the field or in the collections we visited. However, a single female of *Xylocopa macrops* deposited at MACN and labeled “Paraná, Noviembre, n° 190”, was erroneously identified as *Xylocopa subcyanea*. Likewise, the two female specimens recorded by Torreta et al. (2010) as *Xylocopa subcyanea* (deposited at FAUBA) turned out to be *Xylocopa macrops*. Thus, it appears that records of this species for Argentina are misidentified specimens. We examined the type of *Xylocopa subcyanea* currently deposited at MNHN and also examined specimens of this species from Brazil. The female of *Xylocopa subcyanea* can be distinguished from that of *Xylocopa macrops* by the upper gena densely punctate (largely impunctate in *Xylocopa macrops*), disc of clypeus flat, uniformly punctate (largely impunctate and depressed basally in *Xylocopa macrops*), punctures of terga elongate, dense (punctures circular and sparse in *Xylocopa macrops*), and wings subhyaline, brownish (dark brown to black in *Xylocopa macrops*).

**Figures 45–48.**
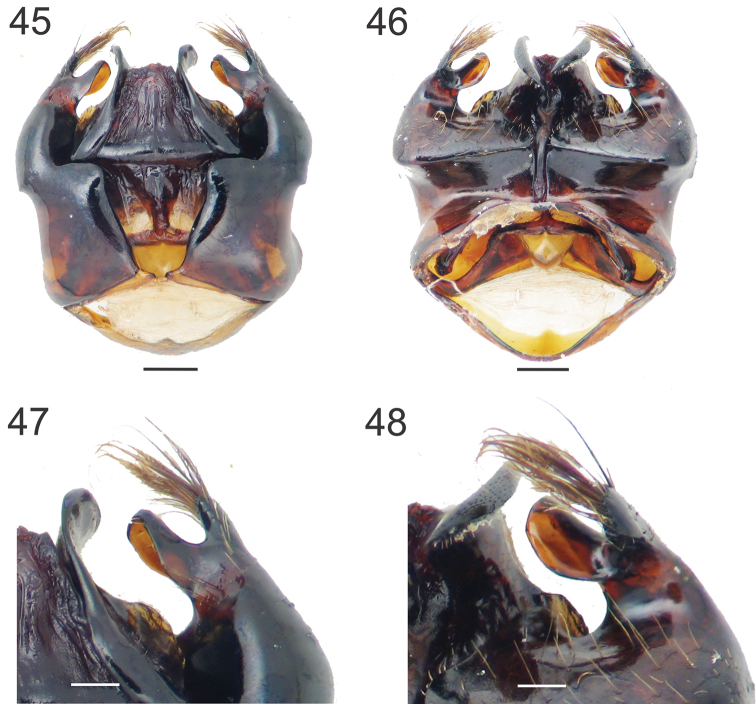
Male genital capsule of *Xylocopa simillima*. **45, 46** dorsal and ventral views respectively **47, 48** detail of the apex of gonostylus and penis valve in dorsal and ventral views. Scale bars: 0.5 mm

**Figures 49–52.**
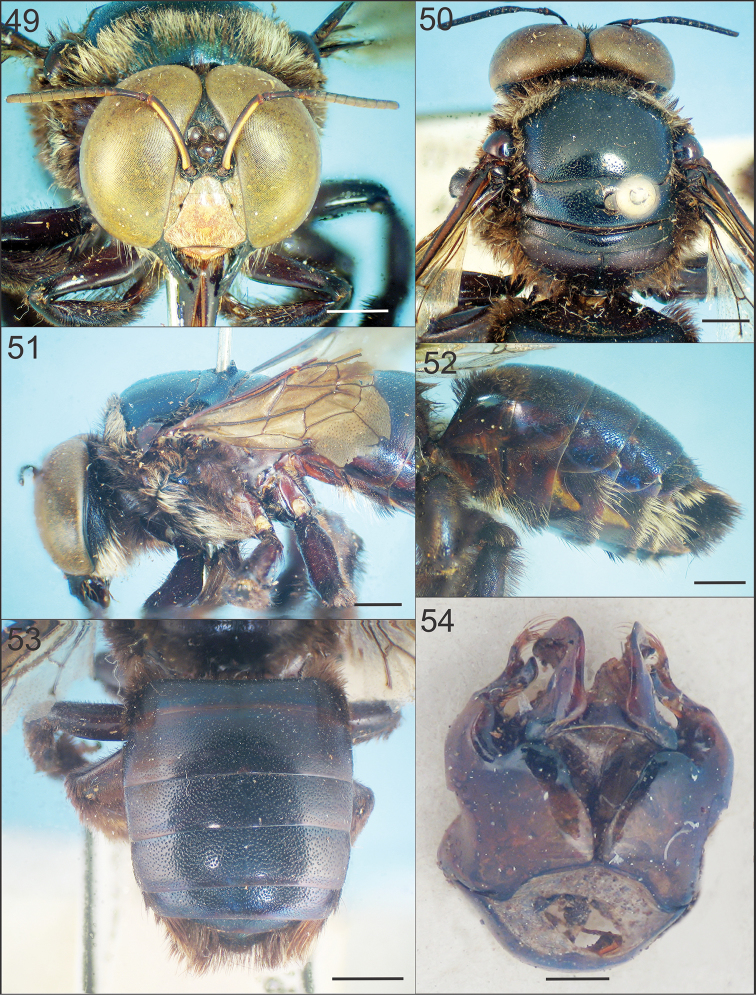
Male holotype of *Xylocopa boops*. **49** Facial view **50, 51** Head and mesosoma in dorsal and lateral views **52, 53** Metasoma in lateral and dorsal views **54** genital capsule in dorsal view. Scale bars: 2 mm, 0.5 mm (Fig. **54**).

**Figures 55–58.**
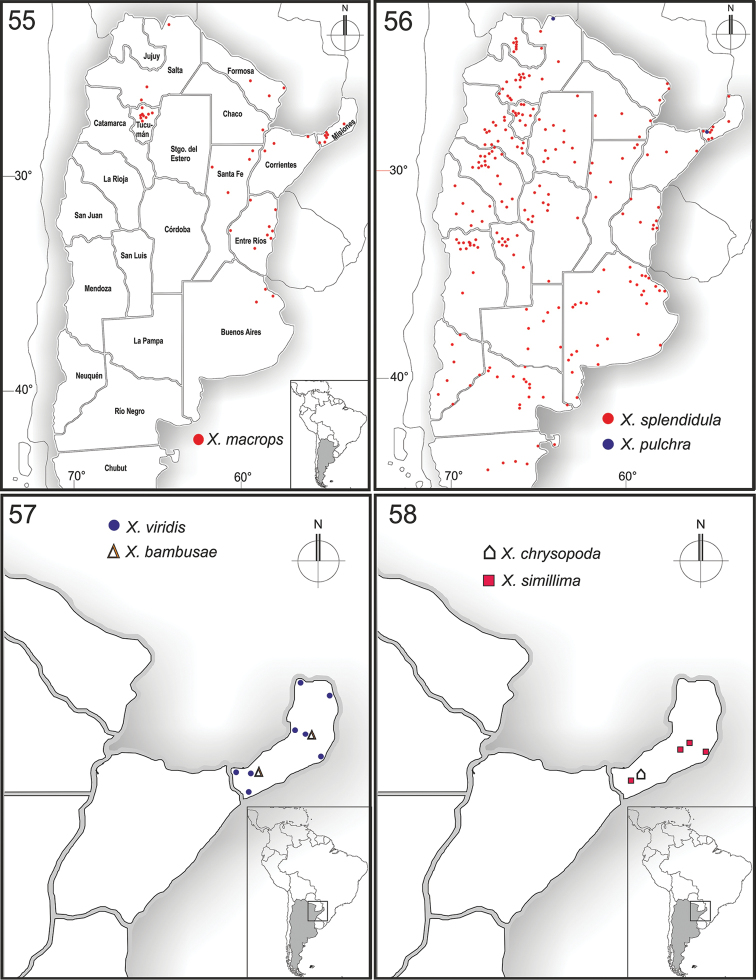
Collection localities for species of Xylocopa subgenus Schonnherria in Argentina.

##### Xylocopa pulchra

Taxon classificationAnimaliaHymenopteraApidae

Smith, 1854

Figures 3 , 15 , 58

Xylocopa pulchra Smith, 1854: 361 (holotype: BMNH 17B.188; ♀, Rio, Brazil) (examined)
Xylocopa (Schonnherria) pulchra : Hurd and Moure 1963: 123.

###### Diagnosis.

This species is known only from the female. It can be easily recognized by the following combination of characters: small body size (body length 15–17 mm); upper gena and vertex densely punctate, punctures separated by at most a puncture width; wings subhyaline, yellowish; metasoma dark brown to black with weak blue-green metallic highlights; and discs of T2–T5, each with a broad, medially interrupted apical band of dense yellowish setae (Fig. 15).

###### Female.

Body length 16 (15.3–16.7); head length 4.1 (4.0–4.2); head width 5.1 (5.0–5.2); mesosoma width 5.7 (5.6–5.8); metasoma width 6.1 (6.0–6.2); forewing length 11.3 (10.7–12.0); forewing width 3.2. *Coloration.* Integument dark brown to black including tegula, with weak blue-green higlights on metasoma. Wings subhyaline with weak golden highlights, veins yellowish-brown. *Pubescence.* Dark brown to black, except: anterior surface of metatibia distally, outer surface of metabasitarsus, and sides of S2–S4 with whitish setae; metabasitarsus externally with pale hairs; discs of T2–T5 each with a broad, medially interrupted apical band of dense yellowish setae (Fig. 15). Mesosoma with abundant plumose setae except on discs of mesoscutum and mesoscutellum. *Sculpturing.* Weakly imbricate to smooth and shiny. Vertex and upper gena densely punctate, punctures separated by at most a puncture width. Mesoscutum with punctures separated by 1–2 times a puncture width, smooth and shiny on disc; mesoscutellum with punctures smaller and sparser than those on mesoscutum, except smooth and shiny on disc. Metasomal terga with circular to ovoid punctures separated by 1–2 times a puncture width, punctures denser laterally and on apical terga; sterna impunctate mediolongitudinally with punctures sparser than on terga. *Structure*. Head broader than long (1.2–1.3:1); proportion of upper to lower interocular 1:1; middle interocular distance 3.2–3.6; vertex broad, distance between median ocellus and posterior margin of vertex 4.0–4.5 OD; orbitoccipital distance short, 0.5–0.7; lateral ocelli on supraorbital line; interocellar distance to ocelocular distance 0.9–1:1, interocellar to ocelloccipital 0.8:1; ocellocular to alveolocellar 0.7:1; orbitoccipital to ocellocular 0.7–0.9:1; alveolocular to interalveolar 1.1–1.2:1; clypeocellar distance to distance between median ocellus and posterior margin of vertex 1.2:1; frontal carina low; clypeus broader than long (2.2–2.3:1); proportion of length of scape, pedicel and F1–F3: 3.9:0.4:1: 0.5:0.5:0.6, respectively; labrum broader than long, with three basal protuberances, median tubercle distinct, longitudinally elongate, sublateral tubercles small, obscured by pubescence. Mesoscutellum gently convex; metanotum and propodeum subvertically inclined.

###### Male.

Unknown

###### Distribution.

Hurd (1978: 23) listed this species from Brazil, Paraguay, possibly Colombia, and the provinces of Misiones and Salta in Argentina (Fig. 58). It occurs in the Paranaense biogeographic province (see comments below).

###### Comments.

*Xylocopa pulchra* is known only from the female, and the male of *Xylocopa chrysopoda* may be conspecific with this species, as suggested by Schrottky (1902). As for *Xylocopa bambusae* and *Xylocopa chrysopoda*, this species is rare in collections, and it has not been captured in the last 65 years. In addition to the type, we were only able to examine a historical specimen from Misiones deposited at MLP and specimen from Pocitos, Salta, deposited at USNM. The presence of this species in Colombia needs to be confirmed (Gonzalez et al. 2009).

##### Xylocopa simillima

Taxon classificationAnimaliaHymenopteraApidae

Smith, 1854

Figures 4 , 10 , 16 , 22 , 45–48 , 58

Xylocopa simillima Smith, 1854: 357 (holotype: BMNH 17B.196; ♀, Australia) (examined)
Xylocopa mendax Maidl, 1912: 319 (syntype: NMW; ♀, Rio Grande, Brazil); Hurd and Moure 1961: 191 (synonym with *simillima*)
Xylocopa rotundiscuta Brèthes, 1916: 418 (lectotype: MACN; ♀, Misiones, Argentina) (examined); Hurd and Moure 1961: 191 (lectotype designation and synonym with *simillima*)
Xylocopa (Schonnherria) simillima : Hurd and Moure 1963: 123.

###### Diagnosis.

The female of this species can be distinguished from other Argentinean species of *Schonnherria* by the following combination of characters: large body size (body length 20–23 mm); labrum basally with a distinct, large, single capitate tubercle about as large as median ocellus; integument dark brown to black throughout, with basal three or four terga often with distinct broad, light reddish brown bands on discs (rarely orange) (Fig. 16); metasomal terga with punctures circular to ovoid, sparsely punctate on discs of basal terga (2–4 times a puncture width), punctures denser laterally and on apical terga; discs of basal terga with very short, black, simple setae, each seta barely exiting puncture, setae increasing in length laterally and on apical terga. The male can be easily recognized by the combination of compound eyes strongly convergent dorsally (Fig. 10), metasomal terga dark brown to black with basal terga light reddish brown on discs, and wings dark brown.

###### Female.

Body length 21.8 (20.3–23.0); head length 5.8 (5.5–6.1); head width 6.6 (6.4–7.0); mesosoma width 7.5 (7.2–8.4); metasoma width 9.1 (8.8–9.6); forewing length 19.6 (18.6–19.7); forewing width 5.7 (5.6–6.2). *Coloration.* Integument black with posterior margin of mesoscutellum and basal three or four metasomal terga with broad, light reddish brown bands on discs (terga rarely orange). Wings dark brown with violet highlights. *Pubescence.* Dark brown to black. Head with scattered setae on vertex, gena, paraocular area, and clypeus distally. Mesosoma with abundant, plumose setae except nearly asetose on disc of mesoscutum and posterior half of mesoscutellum. T1 with scattered plumose setae on basal half; distal half of T1, as on discs of T2–T5, with very short (0.2–0.3 times OD), appressed simple setae, each barely exiting puncture, setae becoming longer and denser towards apical terga; disc of T6 and sides of T1–T6 each with long, semierect, simple and plumose setae; sterna with semierect, long setae as long as those on sides of terga. *Sculpturing.* Weakly imbricate to smooth and shiny. Vertex and upper gena largely impunctate, punctures denser behind lateral ocelli. Mesoscutum with punctures slightly smaller and denser than those on vertex on anterior margin and lateral to parapsidal lines, impunctate otherwise; mesoscutellum largely impunctate, punctures separated by at least two times a puncture width. Terga with circular to ovoid punctures as large and coarse as those on mesoscutellum, separated by 1–2 times a puncture width, denser laterally on each tergum and towards apical segments; sterna with punctures smaller, nearly contiguous laterally, larger and sparser medially. *Structure.* Head broader than long (1.1–1.2:1); compound eyes with inner margins parallel; proportion of upper to lower interocular distance 0.8–0.9:1; middle interocular distance 4.2–4.5; vertex broad, distance between median ocellus and posterior margin of vertex 4.6–5.3 OD; orbitoccipital distance short 1.1–1.3; lateral ocelli below supraorbital line; interocellar distance to ocelocular distance 0.6:1; interocellar to ocelloccipital 0.3–0.4:1; ocellocular to alveolocellar 0.7–0.9:1; orbitoccipital to ocellocular 1.0–1.3:1; alveolocular to interalveolar 1.2–1.4:1; clypeoalveolar distance about 1.8 times longitudinal diameter of antennal socket; clypeocellar distance to distance between median ocellus and posterior margin of vertex 0.7–0.9:1; frontal carina moderately elevated, forming a small tubercle just above lower tangent of anntenal sockets; clypeus broader than long, 2.2–2.5:1; proportion of length of scape, pedicel and F1–F4: 2.9–3.2:0.2–0.4: 1:0.3–0.4:0.4–0.5:0.4–0.5, respectively; labrum basally with a distinct, large, single capitate tubercle about as large as median ocellus. Mesoscutellum gently convex; metanotum and propodeum vertical in profile.

###### Male.

As in the female, except for usual secondary sexual characters and as follows: body length 22.6; head length 5.7; head width 6.8; mesosoma width 8.8; metasoma width 9.8; forewing length 20.6; forewing width 5.6. *Coloration*. Clypeus, supraclypeal area and lower paraocular area yellow. S*tructure.* Head broader than long (proportion 1.2:1); compound eyes enlarged, strongly converging dorsally (Fig. 10); proportion of upper to lower interocular distance 0.3:1; upper interocular distance 1.6 OD; middle interocular distance 2.3; distance between median ocellus and posterior margin of vertex 4.2 OD; orbitoccipital distance absent due to enlargement of eyes; lateral ocelli below supraorbital line; interocellar to ocelocular distance 4:1; interocellar to ocelloccipital 0.2:1; ocellocular to alveolocellar 0.1:1; orbitoccipital to ocellocular reduced; alveolocular to interalveolar 0.4:1; clypeocellar distance to distance between median ocellus and posterior margin of vertex 0.7:1; clypeus broader than long, 1.7:1; proportion of length of scape, pedicel and F1–F4 2.8:0.4:1:0.2:0.4:0.4, respectively. Genitalia as in Figs 45–48.

###### Distribution.

This species occurs in Misiones (Fig. 58), an area that is included within the Paranaense biogeographic province.

###### Comments.

Brèthes’ original description of *Xylocopa rotundiscuta* was based on three females. To stabilize this name, Hurd and Moure (1961: 191) unambiguously designated one of the syntypes as the lectotype. This specimen, deposited at MACN, has a locality label that reads “Misiones”, a catalogue number 7597 [misinterpreted by Hurd and Moure as 2597], a handwritten number 765 [interpreted by Hurd and Moure as 753], and a lectotype label. Such a valid designation appear to have been missed by Moure (2007: 668).

##### Xylocopa splendidula

Taxon classificationAnimaliaHymenopteraApidae

Lepeletier de Saint Fargeau, 1841

Figures 5 , 11 , 17 , 23 , 28 , 33 , 38 , 43 , 56

Xylocopa splendidula Lepeletier de Saint Fargeau, 1841: 190 (holotype: ♀, Brazil, whereabouts unknown, see comments below)
Xylocopa (Schonnherria) splendidula : Hurd and Moure 1963: 123.

###### Diagnosis.

Both sexes of this species can be distinguished easily from other Argentinean species of *Schonnherria* by the following combination of characters: small to medium body size (body length 13–19 mm); body pubescence entirely black in the female, in the male with dense off-white pubescence on mesepisternum, tegula anteriorly, sides of mesoscutum and mesoscutellum, and dorsum of T1; meso- and metasoma with distinct metallic blue highlights; wing membrane subhyaline with weak violet highlights; male compound eyes enlarged, dorsally convergent, with upper margins separated by at least 2.7 times OD (Fig. 11); and hind leg of male unmodified, except by metatibia with long, slender subapical projection on inner margin, similar in size and thickness to tibial spur. This species can be confused with *Xylocopa macrops*, particularly the male, by the compound eyes distinctly convergent dorsally, metasoma with metallic blue highlights, off-white pubescence on mesosoma and T1, and wings subhyaline. However, in *Xylocopa macrops* the compound eyes are larger and closer dorsally (0.54–0.72 times OD) than in *Xylocopa splendidula* and the hind leg is distinctly modified, with coxa and trochanter each bearing a tooth or spine, femur strongly protuberant ventrally, tibia with two large subapical spines on its inner margin, and basitarsus robust, shorter than the tibia (slender and longer than tibia in *Xylocopa splendidula*). In addition, the labrum is yellow and the metasomal sterna are maculated in *Xylocopa macrops* (labrum and metasoma inmaculate in *Xylocopa splendidula*).

###### Female.

Body length 17.2 (16.6–18.3); head length 4.9 (4.7–5.0); head width 5.5 (5.2–5.8); mesosoma width 6.3 (5.8–6.6); metasoma width 7.1 (6.6–7.5); forewing length 13.3 (12.7–14.0); forewing width 3.7 (3.6–3.8). *Coloration*. Integument dark brown to black with distinct metallic blue highlights, particularly dorsum of meso- and metasoma. Wings subhyaline with weak violet highlights. *Pubescence.* Dark brown to black, except patch of off-white setae on sides of T5. Head with abundant setae, scattered on gena and vertex. Mesosoma with abundant, plumose setae except nearly asetose on disc of mesoscutum and anterior half of mesoscutellum. T1 with scattered plumose setae on basal half; distal half of T1, as on discs of T2–T5, with very short, appressed simple setae, each barely exiting puncture, setae becoming longer and denser towards apical terga; disc of T6 and sides of T1–T6 each with long, semierect, simple and plumose setae; sterna with semierect, long setae as long as those on sides of terga. *Sculpturing.* Weakly imbricate to smooth and shiny. Vertex and upper gena with punctures separated by 1–2 times a puncture width, punctures scattered on upper gena. Mesoscutum with punctures separated by 1–2 times a puncture width, punctures becoming sparser towards center to completely absent on disc; mesoscutellum largely impunctate, with few, scattered punctures. Metasomal terga with circular to ovoid punctures separated by 1.0–1.5 times a puncture width, punctures becoming denser laterally and towards apical terga; sterna with elongate punctures on discs, punctures contiguous laterally, becoming sparser medially. *Structure.* Head broader than long (1.1–1.2:1); compound eyes with inner margins parallel; proportion of upper to lower interocular distance 0.8–0.9:1; middle interocular distance 3.4–4.0; vertex broad, distance between median ocellus and posterior margin of vertex 3.7–4.4 OD; orbitoccipital distance 0.6–0.8; lateral ocelli below supraorbital line; interocellar distance to ocelocular distance 0.9–1:1; interocellar to ocelloccipital 0.5–0.6:1; ocellocular to alveolocellar 0.6–0.8:1; orbitoccipital to ocellocular 0.9–1.1:1; alveolocular to interalveolar 1.0–1.3:1; clypeoalveolar distance 1.3 times longitudinal diameter of anntenal socket; clypeocellar distance to distance between median ocellus and posterior margin of vertex 0.8–1:1; frontal carina moderately elevated, forming a small protuberance at level of lower tangent of anntenal sockets; clypeus broader than long, 2.1–2.3:1; proportion of length of scape, pedicel and F1–F4: 3.2–3.5:0.3–0.5:1:0.3–0.4:0.4–0.5:0.4–0.5, respectively; labrum broader than long, with three basal protuberances, median tubercle distinct, longitudinally elongate, sublateral tubercles small. Mesoscutellum gently convex; metanotum and propodeum subvertically inclined.

###### Male.

As in the female, except for usual secondary sexual characters and as follows: body length 16.1 (13.3–18.6); head length 4.7 (3.8–4.4); head width 4.9 (4.6–5.2); mesosoma width 6.2 (5.6–6.8); metasoma width 6.8 (6.6–7.6); forewing length 12.6 (12.0–13.3); forewing width 3.3 (3.2–3.4). *Coloration.* Clypeus, supraclypeal area, paraocular area, and anterior surfaces of scape and F1 yellow (Fig. 11). *Pubescense.* Off-white pubescence on mesepisternum, tergula anteriorly, sides of mesoscutum and mesoscutellum, and dorsum of T1. *Sculpturing.* Punctures denser than in the female, particularly on metasomal terga. *Structure.* Compound eyes enlarged, strongly converging dorsally, upper orbital margins separated by at least 2.7 times OD; proportion of upper to lower interocular distance 0.4–0.6:1; upper interocular distance 2.0–2.9 times OD; middle interocular distance 1.7–2.0; distance between median ocellus and posterior margin of vertex 2.6–3.1 OD; orbitoccipital distance 0.1–0.2; lateral ocelli below supraorbital line; interocellar distance to ocelocular distance 2.0–4.5:1; interocellar to ocelloccipital 0.3–0.5:1; ocellocular to alveolocellar 0.1–0.2:1; orbitoccipital to ocellocular 0.5–1:1; alveolocular to interalveolar 0.5–0.7:1; clypeocellar distance to distance between median ocellus and posterior margin of vertex 0.9–1.1:1; clypeus broader than long, 1.4–1.6:1; proportion of length of scape, pedicel and F1–F4 2.8–3.1:0.3–0.4:1:0.3–0.4:0.4–0.5:0.4–0.5, respectively. Legs slender, unmodified, except metatibia with long, slender subapical projection on inner margin, similar in size and thickness to tibial spur; mesobasitarsi longer than tibiae. Genitalia as in Figs 28, 33, 38, 43.

###### Distribution.

This species is presumably widespread in South America (Table 1) as well as in Argentina, where it has been found in all provinces except Tierra del Fuego and Santa Cruz (Fig. 56). It occurs in all biogreophic provinces except subantartica. The southernmost collection locality for this species is Altares, Province of Chubut, near parallel 43°.

###### Comments.

Lepeletier de Saint Fargeau (1841) described this species based on a female specimen from Brazil, which is presumably deposited at MNHN as indicated in the original publication. We received a female specimen from the MNHN identified as the type of *Xylocopa splendidula*. The complete label data for this specimen are as follows: “Museum Paris-EY0000001755 // Type ? [red label] // del` emb. del` Uruguay jusquiana missions [handwritten-round label] // splendidula [handwritten]”. Because this specimen was collected in Uruguay, not in Brazil, it is not the true type of *Xylocopa splendidula*. We also examined from the same museum three specimens of *Xylocopa splendidula* from Brazil, but the label data do not agree with those indicated in the original description. Therefore, the whereabouts of the type of *Xylocopa splendidula* are currently unknown.

In specimens of both sexes the color of legs, propodeum, mandibles and tegulae ranged from black to dark brown, and that of the metasoma from greenish to bluish. The presence of tufts of white setae on the sides of the terminal terga is also a variable character.

##### Xylocopa viridis

Taxon classificationAnimaliaHymenopteraApidae

Smith, 1854

Figures 6 , 12 , 18 , 24 , 29 , 34 , 39 , 44 , 57

Xylocopa viridis Smith, 1854: 360 (holotype: BMNH 17B.186; ♂, Rio Tapajós, Pará, Brazil) (examined)
Xylocopa (Schonnherria) viridis : Hurd and Moure 1963: 123.

###### Diagnosis.

Both sexes of this species are easily recognized by the distinctive metallic green highlights on all tagmata, body pubescence yellowish, and metasomal terga uniformly covered by long, appressed simple setae on discs, and with white, tomentose, plumose setae on apical margins laterally (Figs 18, 24).

###### Female.

Body length 16.7 (14–18); head length 4.5 (4.3–4.7); head width 5.1 (4.6–5.4); mesosoma width 5.9 (5.4–6.2); metasoma width 6.5 (6.0–6.8); forewing length 11.7 (11.0–12.3); forewing width 3.1 (3.0–3.2). *Coloration.* Integument dark brown to black with strong metallic green highlights. Wing subhyaline with weak golden highlights. *Pubescence.* Predominantly whitish to yellowish; black or gray setae on face, vertex, upper gena, mesoscutum, mesoscutellum, sides of T2–T5, and most of T6, S5 and S6; ferrugineous setae on inner surface of tarsi. Head and mesosoma with abundant setae, except asetose on discs of mesoscutum and mesoscutellum. Basal half of T1 with scattered, semierect simple setae; distal half of T1 and T2–T6 with appressed setae on discs, each setae at least twice as long as a puncture width, becoming longer towards apical terga, apical margins laterally with dense, white tomentum of appressed plumose setae (Fig. 18); disc of T6 and sides of T1–T6 each with long, semierect, simple and plumose setae; sterna with semierect, long setae as long as those on sides of terga. *Punctation.* Weakly imbricate to smooth and shiny. Vertex densely punctate, punctures separated by at most a puncture width, becoming enlongate and sparser on upper gena. Mesoscutum with punctures separated by 1–2 times a puncture width, punctures becoming sparser towards center to completely absent on disc; mesoscutellum largely impunctate, with few, scattered punctures. Terga densely and uniformly punctate, setiferous punctures elongate, separated by a puncture width or less; sterna with punctures sparser than on terga. *Structure.* Head broader than long (1.1–1.2:1); compound eyes with inner margins parallel; proportion of upper to lower interocular distance 0.9:1; middle interocular distance 3.2–3.5; vertex broad, distance between median ocellus and posterior margin of vertex 3.7–4.6 OD; orbitoccipital distance 0.6–0.8; lateral ocelli below supraorbital line; interocellar distance to ocelocular distance 0.9–1.1:1; interocellar to ocelloccipital 0.6–0.7:1; ocellocular to alveolocellar 0.6–0.7:1; orbitoccipital to ocellocular 1.0–1.2:1; alveolocular to interalveolar 0.9–1.1:1; clypeoalveolar distance about as long as longitudinal diameter of anntenal socket; clypeocellar distance to distance between median ocellus and posterior margin of vertex 0.9–1:1; frontal carina moderately elevated, slightly protuberant at level of lower tangent of anntenal sockets; clypeus broader than long, 2.1–2.2:1; proportion of length of scape, pedicel and F1–F4: 3.5–3.8:0.4:1:0.4:0.5–0.6:0.5–0.6, respectively. Labrum broader than long, with three basal protuberances, median tubercle distinct, longitudinally elongate, sublateral tubercles small. Mesoscutellum gently convex; metanotum and propodeum vertical in profile.

###### Male.

As in the female, except for usual secondary sexual characters and as follows: Body length 15.3 (14.3–16.3); head length 3.7 (3.5–3.8); head width 4.3 (4.0–4.4); mesosoma width 5.6 (5.2–6.0); metasoma width 6.1 (5.6–6.6); forewing length 12.1 (11.3–12.7); forewing width 3.2 (3.0–3.4). *Coloration.* Outer surface of mandible, labrum, clypeus, supraclypeal area, and anterior surfaces of scape, F1 and F2 yellow. Tegula reddish brown. *Pubescence.* Longer and denser than in female. *Structure.* Compound eyes with inner margins slightly converging dorsally; proportion of upper to lower interocular distance 0.8:1; upper interocular distance 4.0–4.6 OD; middle interocular distance 1.9–2.0; distance between median ocellus and posterior margin of vertex 2.4–2.7 OD; orbitoccipital distance 0.2–0.3; interocellar distance to ocelocular distance 1.8–2.2:1; interocellar to ocelloccipital 0.7–1:1; ocellocular to alveolocellar 0.2–0.3:1; orbitoccipital to ocellocular 0.8–1:1; alveolocular to interalveolar 0.6–0.7:1; clypeocellar distance to distance between median ocellus and posterior margin of head 1.2–1.3:1; clypeus broader than long, 1.4–1.6:1; proportion of length of scape, pedicel and F1–F4: 2.8–3:0.4–0.6:1:0.4–0.5:0.5–0.6:0.5–0.6, respectively. Legs slender, unmodified, except metatibia with blunt subapical projection on inner margin, shorter and thicker than tibial spur; mesobasitarsi longer than tibiae. Genitalia as in Figs 29, 34, 39, 44.

###### Distribution.

This species has been recorded from Bolivia, Brazil, Colombia, Costa Rica, French Guiana, Guatemala, Guyana, Mexico, Panama, Paraguay and Peru (Moure 2007). In Argentina, it is known only from the province of Misiones (Fig. 57), which is included within the Paranaense biogeographic province.

###### Comments.

*Xylocopa viridis*, ranging from southern Mexico to Argentina, appears to be composed of multiple species. An appraisal of museum specimens under *Xylocopa viridis* deposited at SEMC from locations across its distribution range revealed considerable variation in body pubescence, punctation, body size, and shape of the apical projection of the inner surface of the male metatibia. A similar case seems to occur in *Xylocopa varians* Smith, a species with metallic green highligths that has also been recorded from Misiones and presumably confused with *Xylocopa viridis*. We studied the male holotype of *Xylocopa viridis* and the female type of *Xylocopa varians*, both from Brazil, as well as specimens of both species from Brazil and other countries in South America. We observed that both sexes of *Xylocopa varians* can be distinguished from those of *Xylocopa viridis* primarily by the terga with black setae on their discs (yellowish in *Xylocopa viridis*) and the upper gena of the female densely punctate, with a narrow impunctate band behind the outer margins of the compound eyes (upper gena sparsely punctate and with broad impunctate band behind outer margins of compound eyes in *Xylocopa viridis*). We found a female and male specimen from Misiones, both identified by P.H. Hurd as *Xylocopa varians*. These specimens are deposited at MACN and SEMC respectively, and their complete label data are as follows: “7596” // Misiones // 766 // Xylocopa varians Smith, P.H. Hurd 59 (MACN); “Misiones, Pindapoy, II-1956 // SEMC 1232909”. Both specimens agree with the characters listed for *Xylocopa viridis* and thus the record of *Xylocopa varians* for Argentina appear to be based on misidentified specimens.

Specimens of *Xylocopa viridis* from Argentina and southern Brazil also seem to be different from those of northen Brazil, particularly in the length of the setae on metasomal terga, the color of tegulae, and the shape of the apical projection on the inner surface of male metatibia. Such differences are suggestive of a distinct species and further studies are needed to determine the species limits of *Xylocopa viridis*.

### Nesting biology of *Xylocopa splendidula* and *Xylocopa viridis*

Four nests of *Xylocopa splendidula* and 10 nests of *Xylocopa viridis* were found in total. Nests of *Xylocopa splendidula* were found in Villa Atamisqui (28°27’ 36”S, 63° 50'53”W, 123 m.a.s.l) in Santiago del Estero and the following three localities in the province of Buenos Aires: Berisso (34°53'12"S, 57°53'41"W, 6 m.a.s.l), Gral. Dorrego (38°52'37”S, 61°27'41"W, 32 m.a.s.l), and Mercedes (34°38'57"S, 59°24'50"W, 39 m.a.s.l). These nests were found inside dead branches of the following plant species: *Syagrus romanzoffiana* (Cham.) Glassman (Arecaceae), *Stetsonia coryne* (Salm-Dyck) Britton & Rose (Cactaceae), *Salix* sp. (Salicaceae), and *Parkinsonia aculeata* L. (Fabaceae). Sometimes nests of Xylocopa (Neoxylocopa) augusti Lepeletier de Saint Fargeau as well as *Xylocopa frontalis* were found in the same branch with nests of this species.

Nests of *Xylocopa viridis* were collected in two localities in the province of Misiones: Loreto (27°20'16"S, 55°31'55"W, 175 m.a.s.l.) and Iguazú (25°40'41"S, 54°26'58"W, 191 m.a.s.l). All nests were found inside dead branches of *Hovenia dulcis* Thunb. (Rhamnaceae). As in *Xylocopa splendidula*, nests of *Xylocopa frontalis* were also found in the same branch with nests of *Xylocopa viridis*.

**Figures 59–62.**
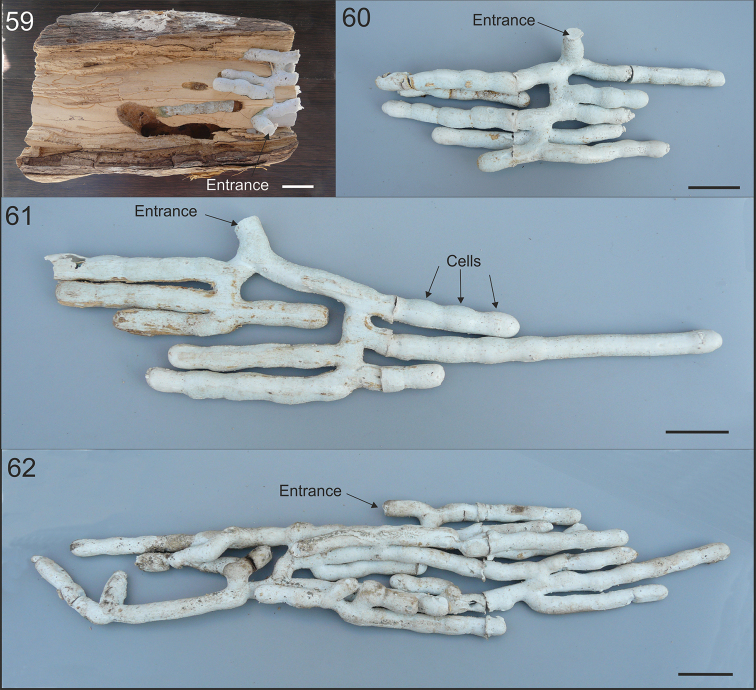
Nests of *Xylocopa viridis* (**59–61**) and *Xylocopa splendidula* (**62**) in Argentina. **59** Section of tree branch showing an opened nest with tunnels filled with silicone rubber **60–62** Extracted molds from tree branch showing arrangement of tunnels and brood cells. Scale bars: 2 cm

As in other species of *Xylocopa* (e.g., Camillo and Garófalo 1982; Gonzalez et al. 2009, Lucia et al. 2014), nests of both species were built inside dead, dry wood, and consisted of a single entrance connected to a system of branched tunnels through a vestibule (Figs 59–62). The tunnels were parallel to the wood grain with the barrel-shaped brood cells built at the end of each tunnel, which were separated from each other by partitions of sawdust. These partitions were thinner in the center, smooth and concave on their outer surfaces, but coarse and flat on their inner surfaces. In both species the length of the tunnel was not significantly correlated with its diameter (Pearson correlation coefficient, *Xylocopa viridis*: *r* = 0.15, *p* = 0.28; *Xylocopa splendidula*: *r* = -0.17, *p* = 0.31), and the number of tunnels per nest was not significantly correlated with the diameter of the branch where the nest was found (*Xylocopa viridis*: *r* = 0.57, *p* = 0.08; *Xylocopa splendidula*: *r* = 0.064, *p* = 0.94). The total number of brood cells varied among nests and species (Table 2) and it was significantly correlated with the total number of tunnels per nest (*Xylocopa viridis*: *r* = 0.96, *p* < 0.05; *Xylocopa splendidula*: *r* = 0.99, *p* < 0.05), but independent of the diameter of the branch (*Xylocopa viridis*: *r* = 0.60, *p* = 0.09; *Xylocopa splendidula*: *r* = 0.07, *p* = 0.93); the number of cells per tunnel was independent of the length of the tunnel (*Xylocopa viridis*: *r* = 0.37, *p* = 0.08; *Xylocopa splendidula*: *r* = 0.13, *p* = 0.46). Dimensions of some features of the nest of both species are indicated in Table 2.

**Table 2.** Measurements (mm) of the nests of *Xylocopa splendidula* and *Xylocopa viridis* from Argentina. Means are given with standard deviation followed by the range and sample size in parenthesis.

Nest feature	*Xylocopa splendidula*	*Xylocopa viridis*
Branch diameter	83.8 ± 40.8 (53.0–142.0, *n* = 4)	113.0 ± 15.6 (95.0–128.0, *n* = 10)
Nest entrance diameter	7.0 ± 0.76 (6.0–8.0, *n* = 8)	7.5 ± 0.76 (7.0–9.0, *n* = 20)
Vestibule	16.2 ± 6.3 (12.0–20.0, *n* = 5)	22.4 ± 2.8 (13.0–26.0, *n* = 9)
No. Tunnels	8.8 ± 10.2 (3.0–24.0, *n* = 4)	5.3 ± 4.6 (1.0–14.0, *n* = 10)
Tunnel diameter	8.8 ± 1.0 (6.9–11.2, *n* = 35)	9.8 ± 0.9 (7.9–12.0, *n* = 53)
Tunnel length	51.7 ± 26.0 (12.0–113.0, *n* = 35)	56.5 ± 24.8 (18.0–153.0, *n* = 35)
Number of cells per nest	18.5 ± 23.8 (4.0–54.0, *n* = 4)	12.6 ± 13.8 (0.0–36.0, *n* = 10)
Cell partition	3.5 ± 0.8 (3.0–5.0, *n* = 15)	4.6 ± 1.1 (2.5–6.0, *n* = 23)
Cell length	12.1 ± 1.4 (10.0–15.0, *n* = 75)	11.4 ± 1.8.8 (9.0–19.0, *n* = 127)

The number of adult females present at the time of collection ranged from one to six in nests of *Xylocopa viridis* (x= 2.3 ± 1.8) and from one to four in nests of *Xylocopa splendidula* (x = 2.7 ± 1.2); adult males were found only in three nests of *Xylocopa viridis* (x = 2.0, 1–3, ± 1.0) and in one nest of *Xylocopa splendidula* (7 males). Females were not dissected to examine the ovarian development or the presence of sperm in their spermatheca. Three females and one male of *Xylocopa viridis* were found dead inside the nest and were parasitized by the conopid fly *Physocephala* sp.; likewise, *Physocephala wulpi* Camras was found parasitizing adults of *Xylocopa splendidula* (Stuke et al. 2011).

## Discussion

The presence of seven of the 11 species of Xylocopa (Schonnherria) recorded for Argentina by Hurd (1978) are confirmed. One of the four remaining species, *Xylocopa boops*, is known only from the type and appears to have been described from a male specimen of *Xylocopa macrops* with unusually large compound eyes. Thus, here it is interpreted as synonym of that species (see species account). The remaining three species, *Xylocopa dimidiata*, *Xylocopa subcyanea*, and *Xylocopa varians*, occur in Brazil and Paraguay and were recorded from Misiones, a province that shares borders with both countries. We did not find any specimens of these species collected in Argentina nor did we capture any of them during the field surveys in Misiones or in any other province of the country. However, we examined a few female specimens of *Xylocopa simillima*, *Xylocopa macrops*, and *Xylocopa viridis* from Misiones that had been identified under those names (see species account). Thus, such records of *Xylocopa dimidiata*, *Xylocopa subcyanea*, and *Xylocopa varians* for Argentina seem to have been misidentified specimens. Yet, we still do not rule out entirely the possibility that these species existed or still exist in Misiones because of the proximity of this province to Brazil and Paraguay, and because three other species, namely *Xylocopa bambusae*, *Xylocopa chrysopoda*, and *Xylocopa pulchra*, were collected in Misiones during the early 1900’s and have not been collected since.

Only two of the seven species of Xylocopa (Schonnherria) occurring in Argentina are widely distributed in the country. *Xylocopa splendidula* exhibits the widest range, found everywhere except in Tierra del Fuego and Santa Cruz (Fig. 56), while *Xylocopa macrops* occurs across the northwest, from Tucumán to Jujuy, and across the east, from northern Buenos Aires to Misiones (Fig. 55). Both species are abundant and frequently found in open fields and urban habitats. The remaining species have been recorded only from Misiones (Figs 57, 58). These species appear to occur across the Paranaense biogeographic province or Interior Atlantic Forest, an ecoregion that consists mostly of rainforests and encompases Misiones, eastern Paraguay, and southeastern Brazil (Cabrera and Willink 1980; Giraudo et al. 2003). Thus, Misiones seems to be at the border of their distribution.

In this study we were able to recognize a number of structural characters in both sexes that seem reliable in species recognition, such as size, shape, density, and distribution of punctures, length, density and type of setae, male sexual secondary modifications (projections of legs, particularly on inner surface of metatibia), and features of the male genitalia. Althought some of these characters have been mentioned by some authors, to date, an emphasis has been made to use patterns of coloration of wings and pubescence (e.g., Schlindwein et. al. 2003). We have made an effort to document and illustrate these structural characters and to them in the keys as well as in the diagnoses and descriptions. Distinctive characters of the genital capsule of the male include the shape of the medial margin of the gonocoxite in dorsal view (Figs 25–29), shape of the ventroapical plate (Figs 30–34), thickness of the penis valve and shape of the lateral carina of the penis valve, and pubescence of the apex of the gonostylus (Figs 35–44, 47, 48). For example, in *Xylocopa splendidula* the ventroapical plate has a well developed, nearly digitiform posteromedial lobe (Figs 33, 43) whereas it barely projects in *Xylocopa chrysopoda* (Figs 31, 41); in *Xylocopa macrops* the penis valve, in dorsal view, is robust and basally broad (Fig. 37) whereas it is slender across its length in *Xylocopa viridis* (Fig. 39); in *Xylocopa chrysopoda* the lateral carina of the penis valve is short and projects into a lobe (Fig. 36) whereas in *Xylocopa splendidula* it is longer, extending towards the apex of the penis valve (Fig. 38); in *Xylocopa viridis* both lobes of the apex of the gonostylus are densely covered by long, plumose setae (Figs 39, 44) whereas in *Xylocopa macrops* the inner lobe is asetose and the outer is sparsely covered by simple setae (Figs 37, 42). These characters may also prove to be useful in future phylogenetic analyses.

The nesting biologies documented here for *Xylocopa splendidula* and *Xylocopa viridis* agree with those of other species of the genus that nest in solid wood, including species of *Schonnherria* (e.g., Jörgensen 1909; Bertoni 1911; Strand 1912; Gonzalez et al. 2009; Lucia et al. 2014). In addition to the nesting substrates recorded in this work, nests of *Xylocopa splendidula* have been found in dead wood of the following species: *Populus piramidalis* (Salicaceae), *Arundo donax* (Poaceae), *Salix* sp. (Salicaceae), *Populus* sp., *Melia azedarach* (Meliaceae), and *Broussonetia papyrifera* (Moraceae) (Holmberg 1884; Friese 1908; Jörgensen 1909; Telleria 2000). Such a wide range of plants used as nesting substrates in *Xylocopa splendidula* contrast with that of *Xylocopa viridis*, in which all 10 nests were found using the same plant species (*Hovenia dulcis*, Rhamnaceae). For both species, the number of cells per nest was significantly correlated with the number of tunnels, and both variables were independent of the diameter of the branch; likewise, the number of cells per tunnel was independent of the length of the tunnel. Thus, the observed variation among nests is probably due to ontogenic differences rather than substrate limitations, as suggested for other species (e.g., Camillo and Garófalo 1982). The same ontogenic differences could also explain the variation in the number of adult females found among nests.

The province of Misiones contains the highest diversity of large carpenter bees in Argentina (Lucia et al. 2014) and future conservation efforts as well as comparative bionomic studies on this group should be focused on this area. We do not know if the same pattern of diversity occurs in other groups of bees, but Misiones is an area well known to contain a high diversity of other organisms, including birds (e.g., Rabinovich and Rapoport 1975; Giraudo et al. 2003).

### Key to species of Xylocopa subgenus Schonnherria in Argentina

Females

**Note.** The female of *Xylocopa chrysopoda* is unknown.

**Table:** 

1	Labrum basally with a distinct, large, single capitate tubercle about as large as the median ocellus (Fig. 4); metasomal terga dark brown to black with basal three or four terga often with broad, light reddish brown bands on discs (Fig. 16) [sometimes terga largely orange]; wings dark brown with violet highlights; large bees (body length 20–23 mm)	***Xylocopa simillima* Smith**
–	Labrum basally with a longitudinally elongated median tubercle and two small sublateral tubercles (often obscured by dense setae); color of metasomal terga variable, never orange or with light reddish brown bands; color of wings variable, hyaline to black; small to medium-sized bees (body length 14–22 mm)	**2**
2(1)	Discs of T2–T5, each with a broad, medially interrupted apical band of dense yellowish setae (Fig. 15), integument not visible among setae	***Xylocopa pulchra* Smith**
–	Discs of T2–T5 without broad, medially interrupted apical bands of dense yellowish setae	**3**
3(2)	Mesoscutellum posteriorly and metasomal terga with distinct metallic green or blue highlights; wings hyaline or subhyline with light reddish brown veins	**4**
–	Mesoscutellum posteriorly and metasomal terga dark brown to black without metallic highlights, at most with very weak blue reflections; wing membrane and venation dark brown to black	**5**
4(3)	Metasomal terga with metallic green highlights, densely and uniformly punctate except along midline, setiferous punctures elongate, separated by a puncture width or less; T1–T5 with yellowish setae on discs, apical margins laterally with dense, white tomentum of appressed plumose setae (Fig. 18); upper gena densely punctate, punctures elongate, separated by 1–2 times a puncture width	***Xylocopa viridis* Smith**
–	Metasomal terga with metallic blue highlights, sparsely punctate, setiferous punctures circular or slightly ovoid, separated by 1–3 times a puncture width; discs of T1–T5 with dark brown to black setae, apical margins without tomentum (Fig. 17); upper gena sparsely punctate, punctures circular or slightly ovoid, separated by at least two times a puncture width	***Xylocopa splendidula* Lepeletier de Saint Fargeau**
5(3)	Clypeus flat in profile, dorsolaterally not elevated from adjacent paraocular area; supraclypeal area flat, frontal carina projected into a distinct tubercle just above or at the upper tangent of anntenal sockets (Fig. 1); vertex, behind lateral ocelli, largely impunctate, punctures small and scattered; small bees (body length 14–17 mm) (Misiones, rarely collected)	***Xylocopa bambusae* Schrottky**
–	Clypeus depressed basally on disc, dorsolaterally elevated from adjacent paraocular area; supraclypeal area depressed on disc, laterally elevated from adjacent paraocular area, frontal carina elevated and forming a low tubercle near lower tangent of antennal sockets, continuing dorsally into a low carina (Fig. 2); vertex, behind lateral ocelli, densely punctate, punctures coarse, separated by 1–2 times a puncture width; larger bees (body length 18–22 mm) (widespread in northern Argentina, commonly collected)	***Xylocopa macrops* Lepeletier de Saint Fargeau**

Males

**Note.** The male of *Xylocopa pulchra* is unknown.

**Table:** 

1	Compound eyes strongly convergent dorsally, upper interocular distance less than three times OD (Fig. 9)	**2**
–	Compound eyes not distinctly convergent dorsally, inner margins parallel or nearly so, upper interocular distance at least four times OD (Fig. 7)	**4**
2(1)	Upper interocular distance 0.4 to 0.6 times lower interocular distance; small bees (body length 13–19 mm)	***Xylocopa splendidula* Lepeletier de Saint Fargeau**
–	Upper interocular distance 0.1 to 0.3 times lower interocular distance; larger bees (body length 21–23 mm)	**3**
3(2)	Labrum black (Fig. 10); wings dark brown; metasomal terga dark brown to black with basal three or four terga often with broad, light reddish brown bands; discs of S2–S6 inmaculate	***Xylocopa simillima* Smith**
–	Labrum yellow (Fig. 9); wings hyaline to subhyaline; metasomal terga with metallic blue highlights, without light reddish bands; discs of S2–S6 each with sublateral yellow maculations	***Xylocopa macrops* Lepeletier de Saint Fargeau**
4(1)	Supraclypeal area with a distinct tuft of long, erect, dense setae obscuring integument (Fig. 7); protrochanter with distinc spine; ventral surface of metatrochanter and metafemur basally glabrous, distinctly protuberant; T1–T5 dark reddish brown, each with a distinct, broad, median yellow maculation on disc, sides and distal margins (inmaculate areas) densely covered by long, dense setae (Fig. 19)	***Xylocopa bambusae* Schrottky**
–	Supraclypeal area without a tuft of long, erect, dense setae; protrochanter without spine or tubercle; ventral surface of metatrochanter and metafemur basally pubescent, not distinctly protuberant; T1–T5 inmaculate	**5**
5(4)	Metasomal terga with distinct metallic green highlights, densely and uniformly punctate, setiferous punctures elongate, separated by a puncture width or less; T1–T5 with short (≤ 1.0 OD) setae on discs, apical margins laterally with dense, white tomentum of appressed plumose setae (Fig. 24); sterna with sparse, short setae on discs, not obscuring integument (commonly collected)	***Xylocopa viridis* Smith**
–	Metasomal terga dark reddish brown to black, at most with weak metallic highlights, setiferous punctures circular to ovoid, separated by 1–2 times a puncture width; T1–T5 with distinctly long (≥ 2.0 OD) setae on discs, apical margins without tomentum (Fig. 20); sterna with distinctly long, dense setae partially obscuring integument (rarely collected)	***Xylocopa chrysopoda* Schrottky**

## Supplementary Material

XML Treatment for Schonnherria

XML Treatment for Xylocopa bambusae

XML Treatment for Xylocopa chrysopoda

XML Treatment for Xylocopa macrops

XML Treatment for Xylocopa pulchra

XML Treatment for Xylocopa simillima

XML Treatment for Xylocopa splendidula

XML Treatment for Xylocopa viridis
